# Large‐Scale Perovskite Single Crystal Growth and Surface Patterning Technologies

**DOI:** 10.1002/smsc.202300085

**Published:** 2023-10-18

**Authors:** Jinshuai Zhang, Jiepeng Song, Qing Zhang

**Affiliations:** ^1^ School of Materials Science and Engineering Peking University Beijing 100871 P. R. China; ^2^ Department of Materials Science and Engineering University of Science and Technology of China Hefei 230026 P. R. China

**Keywords:** optoelectronics, patterning, perovskite, single crystals, single-crystal thin films

## Abstract

In the past decade, metal halide perovskite polycrystalline films have witnessed significant advancements in the field of high‐performance optoelectronic devices, including photodetectors, solar cells, light‐emitting diodes, and lasers. Perovskite films with periodic micro/nanoarrays have garnered substantial attention due to their capability not only to improve the efficiency of individual devices but also to hold great promise for future commercialization. Surpassing their polycrystalline counterparts, perovskite single crystals typically exhibit longer carrier diffusion lengths, extended carrier lifetimes, and enhanced carrier mobility due to the absence of grain boundaries and reduced defects, positioning them as promising candidates for both fundamental studies and advanced optoelectronic devices. To this end, significant endeavors have been dedicated to the development of diverse methodologies for synthesizing large‐scale perovskite single crystals, including bulk single crystals and single‐crystal thin films. Furthermore, aiming to integrate the distinctive functionality with single crystals, considerable efforts have been directed toward the design of certain patterns on single‐crystal surfaces. Herein, this review presents recent progress in technologies for the preparation of large‐scale single crystals and the approaches to patterning their surfaces, highlights the unique advantages of each method, and presents their promising advances in various optoelectronic applications as well as the potential challenges.

## Graphical abstract

This review highlights recent progress in large‐scale perovskite single‐crystal fabrication and surface patterning, covering methods for preparing bulk single crystals and single crystal thin films, along with various surface patterning approaches. The review showcases their applications in optoelectronic devices like photodetectors, solar cells, lasers, and so forth. It also addresses challenges and future research directions, advancing perovskite‐based optoelectronics.

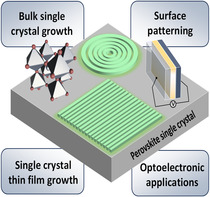

## 1 Introduction

**Figure 1:**
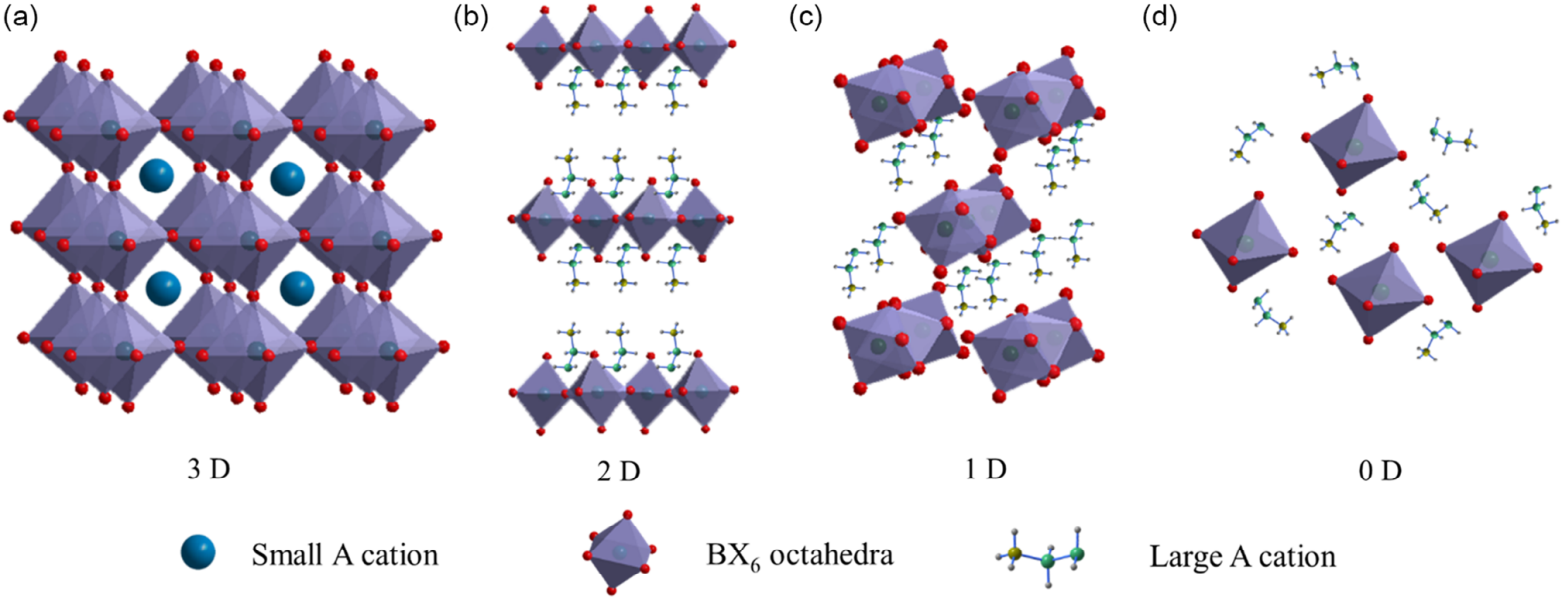
Various dimensionalities of perovskite crystal structure: a) 3D, b) 2D, c) 1D, and d) 0D.

Metal halide perovskites pose a general formula of ABX_3_ or A_2_BX_4_, where A (Cs^+^, Ru^+^, K^+^, CH_3_NH_3_^+^, HC(NH_2_)_2_^+^ or NH_2_CH=NH^2+^) and B (Pb^2+^, Sn^2+^, Eu^2+^ or Mn^2+^) are cations, while X is halide anion (Cl^−^, Br^−^, or I^−^).^[^ ^1^ ^]^ The small A cations, such as Cs^+^, Ru^+^, K^+^, CH_3_NH_3_^+^ (MA^+^), and HC(NH_2_)_2_^+^ (FA^+^) can form a 3D structure with the corner‐sharing BX_6_ octahedra network (**Figure** **1**a). Regarding the larger A cations, such as C_6_H_5_CH_2_CH_2_NH_3_^+^ (PEA^+^), C_4_N_2_H_14_^2+^, and C_8_NH_12_^2+^, their size poses challenges for fitting within the BX_6_ octahedra, resulting in the formation of low‐dimensional (2D, 1D, or 0D) crystal structures (Figure 1b–d). The tuneable crystal structures and variable chemical compositions of perovskites enable the exploration of diverse properties and the realization of multifunctional applications. In 2009, Kojima et al. successfully utilized MAPbBr_3_ and MAPbI_3_ polycrystalline thin films as the active layer in solar cells, achieving a power conversion efficiency (PCE) of 3.8%.^[^ ^2^ ^]^ Since then, significant progress has been made, with certificated PCEs of polycrystalline thin film solar cells surpassing 25%, showcasing their potential for commercial applications.^[^ ^3^ ^]^ This remarkable performance can be attributed to the unique and exceptional optoelectronic properties of metal halide perovskites, including high bipolar carrier mobility,^[^ ^4^ ^]^ high absorption coefficient,[^1^, ^5^] long balanced carrier lifetimes, and long diffusion length,[^4^, ^6^] which promote the development of high‐performance memories,^[^ ^7^ ^]^ photodetectors,^[^ ^8^ ^]^ light‐emitting diodes,^[^ ^9^ ^]^ and lasers.^[^ ^10^ ^]^

Despite extensive efforts to fabricate high‐performance devices, polycrystalline perovskites still face challenges including high defect density, impurities, defect‐induced ion migration, significant nonradiative carrier loss, and environmental instability, leaving room for further improvement.^[^ ^11^ ^]^ Specifically, the high concentration of defects (10^15^–10^16^ cm^−3^), particularly within the grain boundaries (GBs) of polycrystalline perovskite films, traps photogenerated carriers, leading to substantial nonradiative recombination, and consequently imposes severe limitations on device performance.^[^ ^12^ ^]^ Moreover, the presence of oxygen and water molecules along the GBs causes the degradation of films in ambient environments, thereby posing challenges to their long‐term stability.^[^ ^13^ ^]^ Therefore, to fully understand and explore the intrinsic properties of perovskites, the utilization of perovskite single crystals becomes highly desirable. Single crystals devoid of GBs exhibit a substantial reduction in defect density (as low as 10^9^–10^10^ cm^−3^) and trap‐state densities compared to their polycrystalline thin film counterparts, which results in longer carrier diffusion lengths and enhanced carrier mobilities, potentially leading to improve device performance.[^4^, ^14^] Moreover, the absence of GBs in single‐crystal devices prevents the infiltration of oxygen and water molecules, granting them exceptional stability when exposed to external environments.^[^ ^15^ ^]^ In 2012, a pioneering work demonstrated the utilization of the Bridgeman method to prepare CsPbI_3_ bulk single crystal for dye‐sensitized solar cells.^[^ ^16^ ^]^ Subsequently, in a span of 3 years, the development of perovskite single crystals witnessed a significant leap forward with the successful growth of MAPbI_3_ and MAPbBr_3_ bulk single crystals using a simple solution‐processed approach.[^4^, ^17^] This breakthrough sparked a flourishing era in the field. Consequently, a range of bulk single crystals, including 3D CsPbBr_3_, FAPbI_3_, FAPbBr_3_, 2D (PEA)_2_PbBr_4_, 1D [DMEDA]PbBr_4_, and 0D (C_4_H_14_N_2_)_2_In_2_Br_10_, were consecutively fabricated.^[^ ^18^ ^]^ Moreover, to fulfill the requirements of vertical architecture devices, minimize charge carrier transport distances, and reduce nonradiative recombination losses, researchers have dedicated their efforts to the development of single crystal thin films (SCTFs) using various methods. These large‐scale bulk single crystals and SCTFs are opening new avenues for applications such as photodetector arrays,^[^ ^19^ ^]^ X/γ‐ray detectors,^[^ ^20^ ^]^ solar cells,^[^ ^21^ ^]^ and lasers.^[^ ^22^ ^]^

In addition to the advancement in semiconductor quality achieved through using single crystals, pattern engineering has emerged as an effective approach for designing and fabricating tailored morphologies, ultimately enhancing device performance. In 2001, Kagan et al. employed polydimethylsiloxane (PDMS) as a templet to pattern the (C_6_H_5_C_2_H_4_NH_3_)_2_SnI_4_ polycrystalline thin film with a line width of approximately 3 μm, resulting in transistors with minimal current leakage. In recent years, the growing emphasis on high‐performance perovskite optoelectronics has brought increased attention to the advantageous benefits of enhanced optical and electrical properties in the field of patterning. Patterning techniques applied to polycrystalline thin film surfaces have demonstrated improved PCEs in solar cells, enhanced stability and responsibility in photodetectors, and enabled their use as laser resonance cavities and photonic crystals. The combination of patterning technologies and single crystals harnesses the potential to significantly enhance device performance. Remarkable advances in various fields, including lasing, photodetectors, and integrated photonic systems, have been achieved through the utilization of outstanding patterning technologies and devices based on perovskite single crystals.

This review provides an overview of recent advancements in the fabrication of large‐scale perovskite single crystals and surface patterning technologies. Section 2 summarizes the primary methods for preparing bulk single crystals and SCTFs. Section 3 focuses on the various surface patterning technologies used for perovskite single crystals, including both top‐down and bottom‐up approaches. Section 4 briefly discusses the remarkable applications of single crystals and surface‐patterned samples in optoelectronic devices, including photodetectors, solar cells, and lasers. Finally, potential challenges regarding the fabrication of large‐scale single crystals and surface patterning technologies are summarized, providing insights into future research directions.

## 2 Large‐Scale Single Crystal Synthesis Method

### 2.1 Bulk Single Crystal

Bulk single crystals offer a unique opportunity to investigate the intrinsic and fundamental properties of perovskite materials. This section summarizes the strategies and fundamental principles for preparing bulk single crystals. Methods for growing perovskite bulk single crystals can be categorized into two groups based on the growth environment: solution‐processed and solid growth techniques. Wherein, solution‐processed methods encompass inverse temperature crystallization (ITC), lowing temperature‐induced crystallization (LTC), and antisolvent vapor‐diffusion crystallization (AVC), while the Bridgeman method is utilized for perovskite solid growth.

#### 2.1.1 Solution‐Processed Methods

**Figure 2:**
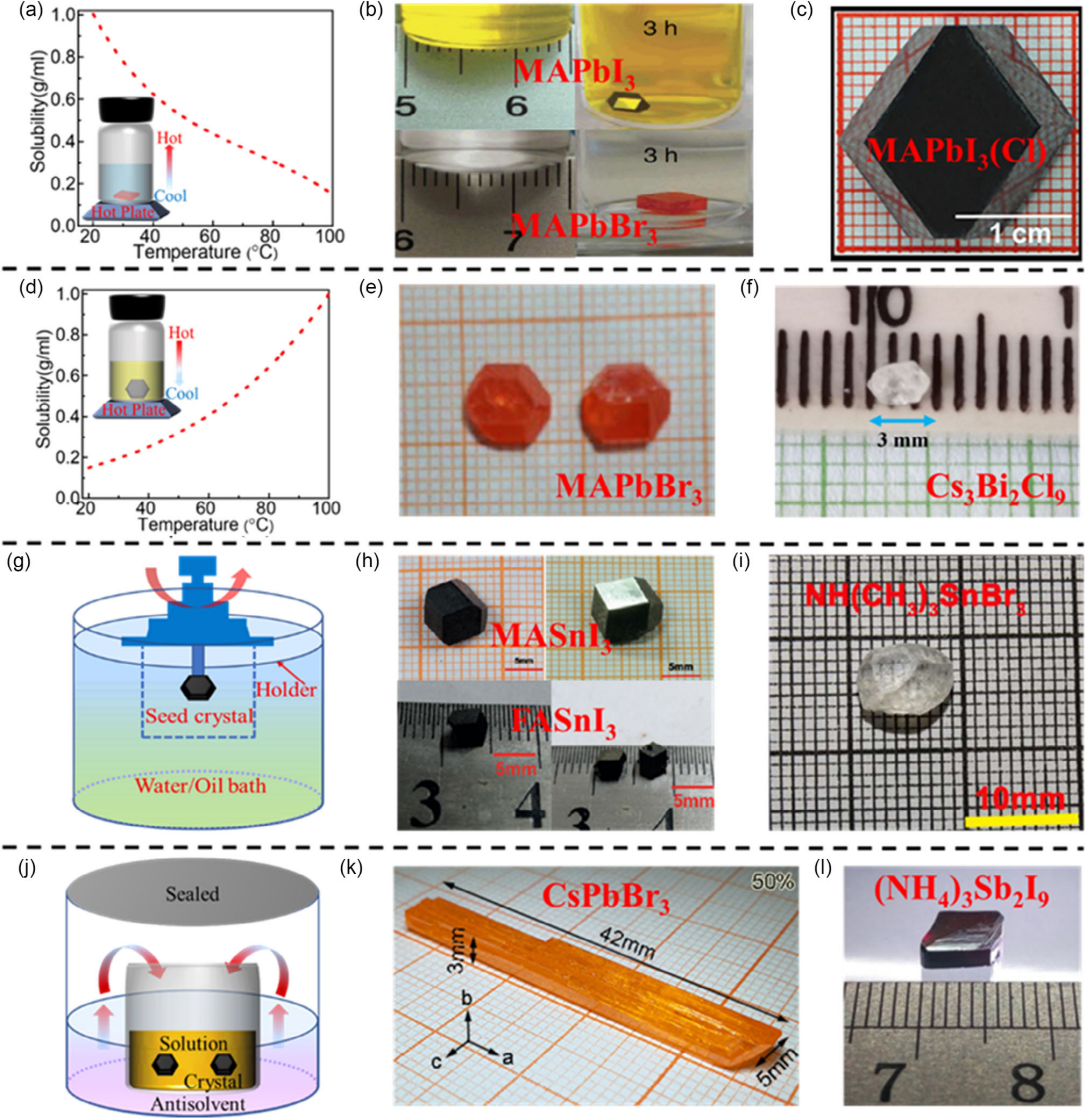
Growth mechanisms and photographs of solution‐processed bulk single crystals. a–c) ITC method for the preparation of single crystals where the solubility of perovskites decreases as the temperature of the solution is gradually increased. b) Reproduced under the terms of the CC‐BY Creative Commons Attribution 4.0 International license (https://creativecommons.org/licenses/by/4.0).^[^ ^26^ ^]^ Copyright 2015, The Authors. Published by Springer Nature. c) Reproduced with permission.^[^ ^27^ ^]^ Copyright 2016, the American Chemical Society. d–f) LTC method for the preparation of single crystals where the solubility of perovskites decreases as the temperature of the solution is gradually decreased. e) Reproduced with permission.^[^ ^33^ ^]^ Copyright 2016, John Wiley and Sons. f) Reproduced with permission.^[^ ^34^ ^]^ Copyright 2021, the American Chemical Society. g–i) TSSG/BSSG method and the resulting single crystals prepared using these techniques. h) Reproduced with permission.^[^ ^35^ ^]^ Copyright 2016, John Wiley and Sons. i) Reproduced with permission.^[^ ^37^ ^]^ Copyright 2016, the American Chemical Society. j–l) AVC method and the photographs of CsPbBr_3_ and (NH_4_)Sb_2_I_9_ single crystals. k) Reproduced with permission.^[^ ^41^ ^]^ Copyright 2017, the American Chemical Society. l) Reproduced with permission.^[^ ^42^ ^]^ Copyright 2017, John Wiley and Sons.

The ITC method exploits the decreased solubility of certain perovskites in specific solvents with increasing temperature (**Figure** **2**a). In 2015, Hagfeldt's group reported a notable decrease in the solubility of MAPbI_3_ in γ‐butyrolactone (GBL) as the solution was heated to high temperatures.^[^ ^23^ ^]^ Similar effects were observed in other systems, such as MAPbBr_3_ in N, N‐dimethylformamide (DMF), and MAPbCl_3_ in dimethyl sulfoxide (DMSO).^[^ ^24^ ^]^ The solubility of these perovskites decreases with increasing temperature and can drop several times as the solution temperature rises from room temperature to 100 °C.^[^ ^25^ ^]^ As the precursor is continuously heated, the solution becomes supersaturated, resulting in the precipitation of perovskite crystals within the solution. Moreover, the ITC method has proven to be highly efficient in producing large‐scale and high‐quality perovskite single crystals. For instance, the size of ITC‐prepared MAPbI_3_ and MAPbBr_3_ crystals can reach 3–5 mm within 3 h (Figure 2b),^[^ ^26^ ^]^ and the crystal size can be further increased by continually feeding fresh solution. Liu et al. fabricated a 2 inch sized MAPbX_3_ single crystal by employing a repeated refresh solution process. By precisely controlling the increasing temperature rate (2 °C day^−1^) to avoid temperature fluctuations and the formation of multiple seed crystals, they successfully harvested high‐quality and large‐sized MAPbBr_3_ single crystals (up to 47 × 41 × 14 mm^3^) at lower temperatures.^[^ ^19^ ^]^ In the pursuit of high‐quality MAPbI_3_ single crystals, Lian et al. used chlorine as a mediator during the solution growth process.^[^ ^27^ ^]^ As a result, they successfully obtained a large‐sized MAPbI_3_ (Cl) single crystal of 20 × 18 × 16 mm^3^ with a low trap density of 7.6 × 10^8^ cm^−3^ (Figure 2c). Additionally, the ITC method can be extended to the synthesis of all‐inorganic perovskites, such as CsPbX_3_. For instance, Dirin et al. prepared the CsPbBr_3_ in DMSO and obtained an 8 mm sized crystal within a few hours.^[^ ^28^ ^]^ Despite its poor solubility, CsPbBr_3_ crystals can also be prepared by dissolving CsBr and PbBr_2_ in high‐temperature DMF (100 °C) and subsequently heating them to 120 °C. By controlling the ratios of CsBr and PbBr_2_ precursors, different phases of CsPbBr_3_, Cs_2_PbBr_5_, and Cs_4_PbBr_6_ can be synthesized.^[^ ^29^ ^]^

In contrast to ITC, the LTC method relies on the decreased solubility of perovskites in HX‐based solvents as the temperature decreases (Figure 2d). In 1987, Poglitsch and Weber reported the growth of MAPbX_3_ single crystals by cooling a solution containing equimolar ratios of PbX_2_ and MAX in an HX aqueous solvent.^[^ ^30^ ^]^ However, when it comes to growing MAPbI_3_ single crystals, it is crucial to maintain the temperature above 40 °C to avoid the formation of the yellow needle‐like phase of MA_4_PbI_6_·2H_2_O.^[^ ^31^ ^]^ Therefore, precise temperature regulation during the growth process is essential to obtain high‐quality crystals and prevent the formation of undesired byproducts. Dang et al. successfully obtained a 10 × 10 × 8 mm^3^ MAPbI_3_ single crystal after about 1 month by carefully adjusting the temperature from 65 °C to slightly above 40 °C to prevent the formation of MA_4_PbI_6_·2H_2_O, indicating that the LTC method is time‐consuming.^[^ ^32^ ^]^ The decreasing‐temperature method is not limited to the growth of MAPbI_3_. As depicted in Figure 2e, a 7 × 7 × 3 mm^3^ MAPbBr_3_ single crystal was successfully grown by gradually cooling and evaporating the HBr‐based solution for more than 10 days.^[^ ^33^ ^]^ Interestingly, compared to single crystals prepared in DMF that exposed the largest crystal face of {100}, the MAPbBr_3_ crystal grown from HBr exhibited the largest crystal face of {110}, suggesting that the choice of solvents can influence the crystal morphology and crystallization habit.

Furthermore, the LTC method has also been adapted for the growth of Pb‐free perovskites. Tailor and Satapathi grew a Cs_3_Bi_2_Cl_9_ single crystal by slowly cooling the solution from 120 °C to room temperature at a rate of 0.3 °C h^−1^, resulting in the formation of a 3 mm single crystal (Figure 2f).^[^ ^34^ ^]^ Additionally, modified methods have been employed to achieve high‐yield and larger single crystals. As schematically illustrated in Figure 2g, the HX‐based solution was utilized in conjunction with either the top‐seeded solution growth (TSSG) or the bottom‐seeded solution growth (BSSG) method, where a seed crystal was affixed to either the top or bottom of the solution container using a Si wafer/Pt wire setup, respectively. Beyond the successful growth of a 10 mm‐sized MAPbI_3_ single crystal using the TSSG method, which benefits from continuous material supply facilitated by convection between the lower and higher temperature regions,^[4a]^ this approach has also been employed for the preparation of several millimeter‐sized Sn‐based perovskites, such as MASnI_3_ and FASnI_3_ single crystals (Figure 2h).^[^ ^35^ ^]^ In contrast, utilizing the BSSG method, Lian et al. obtained a 12 × 12 × 7 mm^3^ MAPbI_3_ single crystal by carefully controlling the gradual temperature reduction process from 373 K to 330 K over 15 days. This was achieved by fixing a small MAPbI_3_ crystal at the end of a Pt wire and immersing it in the solution.^[^ ^36^ ^]^ Similar to TSSG, the BSSG method has also been widely employed for the growth of Pb‐free perovskites, such as NH(CH_3_)_3_SnBr_3_ (Figure 2i) and NH(CH_3_)_3_SnCl_3_ bulk single crystals.^[^ ^37^ ^]^

The AVC method takes advantage of the solubility behavior of perovskites in different solvents. Metal halide perovskites typically exhibit high solubility in GBL, DMF, and DMSO solvents, while exhibiting low or no solubility in solvents such as dichloromethane (DCM), chlorobenzene (CB), benzene, diethyl ether.^[^ ^38^ ^]^ The use of antisolvents was initially applied to the preparation of high‐density and uniform perovskite polycrystalline thin films. In 2014, the Cheng and Seok groups utilized the fast‐deposition crystallization^[^ ^39^ ^]^ and solvent engineering methods^[^ ^40^ ^]^ to fabricate MAPbI_3_ and MAPb(I_1‐*x*_Br_*x*_)_3_ polycrystalline thin films, respectively, where the antisolvents can accelerate the crystallization process during spin‐coating and promote the formation of high‐quality films.

For the preparation of bulk single crystals, the antisolvent vapor slowly diffuses into the precursor until the material reaches saturation, causing the formation of crystals (Figure 2j). After the successful growth of MAPbX_3_ single crystals by Shi et al.,^[38b]^ the AVC method has been widely adopted for synthesizing various all‐inorganic and lead‐free perovskite single crystals. For instance, Zhang et al. utilized DMSO as a solvent and methanol as an antisolvent to produce large‐sized CsPbBr_3_ single crystals, which exhibited high stability and satisfactory quality (Figure 2k).^[^ ^41^ ^]^ In addition, by using the environmentally friendly solvent ethanol and antisolvent chloroform, lead‐free perovskite (NH_4_)_3_Sb_2_I_9_ crystals with sizes up to several millimeters were grown, showcasing their potential for use in hypertoxic photovoltaic applications (Figure 2l).^[^ ^42^ ^]^ Compared to the ITC or LTC methods, the AVC method offers the advantage of gradually changing solution concentration without temperature fluctuations, resulting in single crystals with ordered lattice arrangements and smooth surfaces.

Besides, it should be highlighted that the solution‐processed method is the most used method for preparation of low‐dimensional perovskite single crystals. The synthesis of 2D MAPbBr_3_ single crystal nanostructures was initially reported by Tyagi et al., using a colloidal synthesis method.^[^ ^43^ ^]^ They added MABr, PbBr_2_, and octylammonium bromide (OABr) to a stirring solution of oleic acid (OLA) and 1‐octadecene at 80 °C. The resulting single crystal nanostructures exhibited a blueshift in absorption (≈0.5 eV) compared to the 3D bulk MAPbBr_3_, attributed to quantum confinement effects. Dou et al. fabricated atomically thin single‐crystal 2D (C_4_H_9_NH_3_)_2_PbBr_4_ directly on a substrate using a solution‐processed method.^[^ ^44^ ^]^ After dropping the precursor solution of C_4_NH_9_NH_3_Br and PbBr_2_ in DMF, CB was used to reduce the solubility and promote the crystallization of (C_4_H_9_NH_3_)_2_PbBr_4_. Ultimately, they obtained a single unit cell or a few unit cells thick squared single crystal plate with several micrometers long. By employing this method, the limitations of conventional exfoliation and CVD methods were successfully overcome.^[^ ^18^ ^]^ Stoumpos et al. reported the fabrication of Ruddlesden–Popper 2D perovskite single crystals by cooling the HX‐based solution with various n values from 1, 2, 3, 4, and ∞ of (CH_3_(CH_2_)_3_NH_3_)_2_(CH_3_NH_3_)_*n*‐1_Pb_*n*_I_3*n*+1_.^[^ ^45^ ^]^ The rapid cooling process created numerous nucleation sites, resulting in the formation of a large number of small crystals. Precise regulation of the cooling rate was crucial for achieving large, high‐quality single crystals. Ultimately, crystals with sizes reaching hundreds of micrometers were prepared.^[^ ^45^ ^]^ Numerous other approaches exist for growing large‐sized low‐dimensional single crystals or SCTFs, which will be summarized in the following sections corresponding to their respective methods.

In contrast, the crystallization kinetics in solution‐processed methods, such as ITC, LTC, and AVC, are guided by the principles of classical crystal nucleation and growth theories, which are primarily governed by the rates of nucleation and growth of the resulting crystals formed from the initial solution.^[^ ^46^ ^]^ The nucleation rate holds paramount significance as it directly influences the density and distribution of nuclei, thereby, exerting a substantial impact on the number and the final size of the crystals. In the conventional nucleation process, the formation of nuclei can be thermodynamically described in terms of the total free energy (ΔG), which comprises both the surface free energy (ΔG_S_) and the bulk free energy (ΔG_V_).^[^ ^47^ ^]^ During the process of homogeneous nucleation, ΔG_V_ becomes negative as it represents the driving force for the phase transition of an atom from the liquid to the solid state, resulting in an overall decrease in the system's free energy. Conversely, ΔG_S_ becomes positive as the formation of new nuclei, which acts as a resistance force during the phase transition, leading to an increase in the system's free energy.^[46a]^ Subsequently, the nuclei grow into larger crystals through two mechanisms: diffusion of monomers from the solution to the surface of the nuclei and surface reactions of the monomers on the nuclei.^[^ ^47^ ^]^

#### 2.1.2 Bridgeman Method

The Bridgeman method, a classic and well‐established technique, has been widely employed for the preparation of large‐sized, dimensionally‐tolerant single crystals since the last century.^[^ ^48^ ^]^ Unlike solution‐processed methods, this method allows for the growth of high‐quality bulk single crystals directly from high‐temperature melts, utilizing high‐purity raw materials without solvent affection. Specifically, the Bridgeman method involves carefully encapsulating the raw materials within a vacuum‐sealed quartz ampoule which is then heated to their specific melting temperatures. At a precise temperature threshold, nucleation commences at the tip of the ampoule and spreads throughout the molten material. Through successive rounds of nucleation, elongated single crystals with rod‐like or columnar shapes are formed, exhibiting impressive sizes.^[^ ^48^ ^]^ In 2012, Chuang et al. utilized the Bridgeman method to prepare a centimeter‐sized CsSnI_3_ single crystal by offering a stable growth environment for Sn‐based perovskite.^[^ ^16^ ^]^

**Figure 3:**
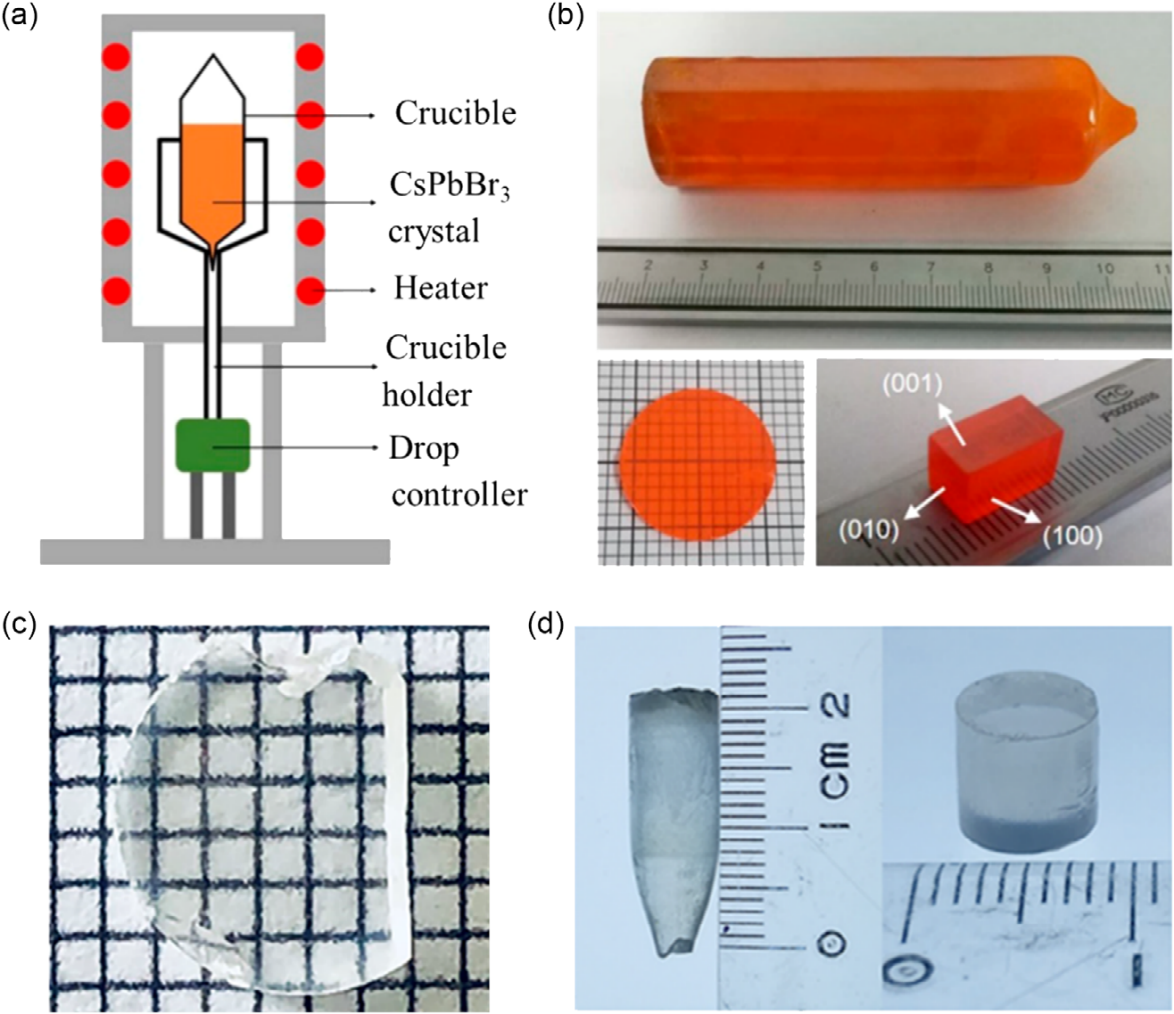
The mechanism of the Bridgeman method and photographs of as‐prepared bulk single crystals. a) Schematic of vertical Bridgman furnace used to grow single crystals. b) Photographs of the prepared large‐volume CsPbBr_3_ single crystal and the polished crystals. a,b) Reproduced with permission.^[^ ^49^ ^]^ Copyright 2018, the American Chemical Society. c) Photograph of 1D CsCu_2_I_3_ single crystal. Reproduced with permission.^[^ ^51^ ^]^ Copyright 2018, the American Chemical Society. d) Photograph of 0D Cs_3_Cu_2_I_5_ single crystal. Reproduced with permission.^[^ ^52^ ^]^ Copyright 2020, John Wiley and Sons.

Recently, the Bridgeman method has been extended to the growth of CsPbBr_3_ single crystals.^[^ ^49^ ^]^ As depicted in **Figure** **3**a, polycrystalline CsPbBr_3_ powder was initially prepared by heating a mixture of CsBr and PbBr_2_ in a quartz ampoule at 600 °C for 24 h under vacuum. The resulting CsPbBr_3_ powder was further refined by removing impurities and repeating this vertical growth process. Subsequently, this high‐purity CsPbBr_3_ was resealed in a quartz ampoule and transferred to the furnace to prepare the single crystal. Finally, an ultra‐large, crack‐free, rod‐shaped CsPbBr_3_ single crystal was achieved, measuring *ϕ* 2.4 cm × 9.0 cm. Through optimization of cutting and polishing techniques, CsPbBr_3_ crystal wafers and rectangular crystals were also obtained (Figure 3b).^[^ ^49^ ^]^ Similar or modified Bridgeman methods have also been adopted by other groups to prepare high‐quality and large‐scale CsPbBr_3_ single crystals.^[^ ^50^ ^]^

Beyond CsPbBr_3_, the Bridgeman method has been employed for the fabrication of low‐dimensional perovskite single crystals as well. For instance, utilizing this method at a low starting temperature of 383 °C, a nonhygroscopic transparent‐color 1D CsCu_2_I_3_ bulk single crystal with uniform dimensions of approximately 7 mm in length and 1 mm in thickness was prepared (Figure 3c).^[^ ^51^ ^]^ Moreover, a centimeter‐sized 0D Cs_3_Cu_2_I_5_ single crystal was also successfully prepared using the vertical temperature gradient Bridgeman method (Figure 3d).^[^ ^52^ ^]^ Although the Bridgeman method has proven effective in producing high‐quality and large‐scale single crystals, its applicability is still limited to all‐inorganic perovskites. Meanwhile, the expensive synthesis process involving vacuum technology and high temperatures hinders its commercial applications.

### 2.2 Single Crystalline Thin Films

Bulk single crystals are invaluable for studying the fundamental properties of perovskite materials, while for vertical devices and integration applications, high‐quality perovskite SCTFs with controllable thickness are highly desired to balance the absorption depth and carrier‐diffusion length. For example, high‐energy ray detection typically necessitates single crystals with several millimeters of thickness to enable the efficient penetration of such rays. In contrast, for solar cells and UV–vis–NIR light photodetection, reducing the thickness of single crystals can enhance charge‐carriers extraction and improve overall device performance. Hence, perovskite single crystals with appropriate thickness, ranging from several millimeters to sub‐micrometers, are essential to fulfill the requirements of different application purposes. This section summarizes the approaches for fabricating perovskite SCTFs, including top‐down synthesis, space‐confined, surface tension assistant, and chemical vapor deposition (CVD) methods.

#### 2.2.1 Top‐Down Synthesis

**Figure 4:**
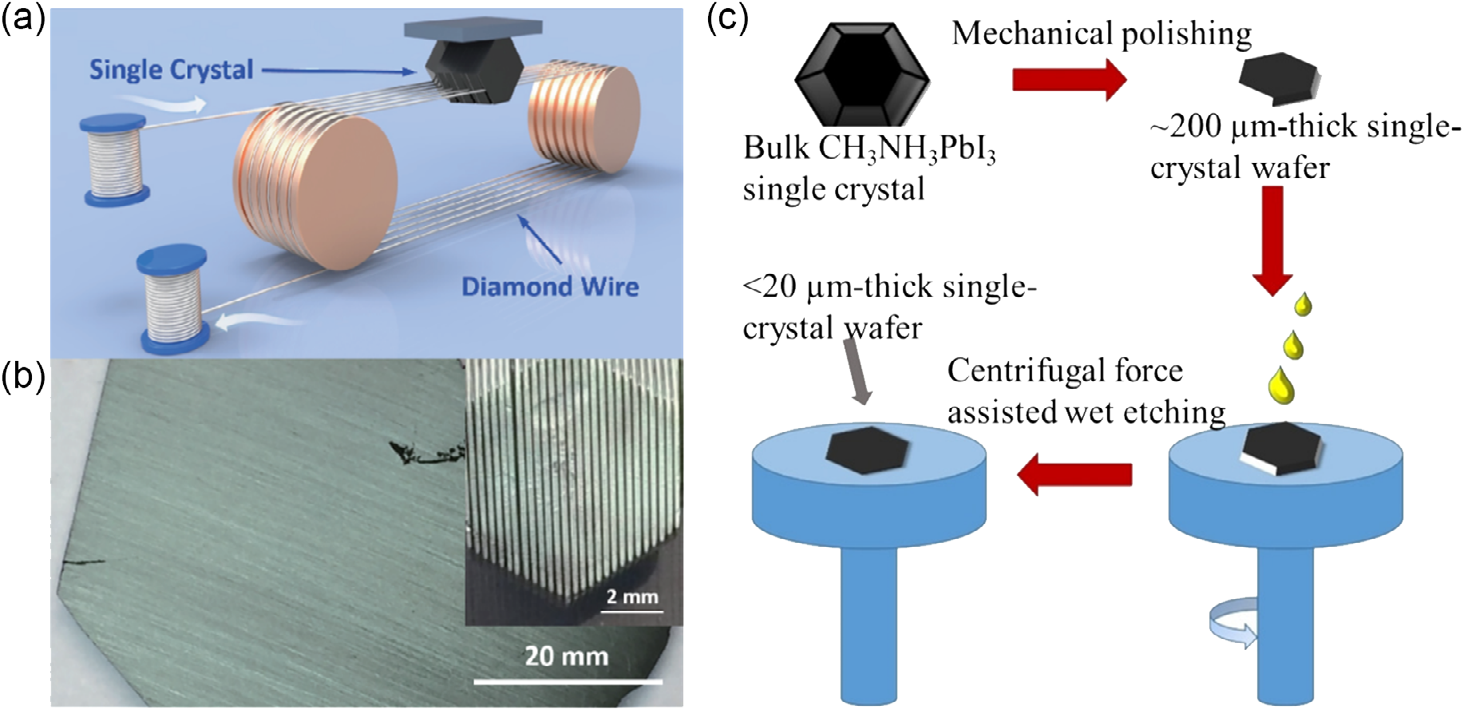
Top‐down methods for perovskite SCTFs preparation. a) Schematic of the single crystal slicing process. b) Photograph of the MAPbI_3_ single crystal wafer and slice. a,b) Reproduced with permission.^[54a]^ Copyright 2017, Springer Nature. c) Schematic illustration of the combined top‐down methods to fabricate MAPbI_3_ SCTFs. Reproduced with permission.^[^ ^56^ ^]^ Copyright 2020, John Wiley and Sons.

Inspired by the traditional method of constructing semiconductor SCTFs using slicing/etching in silicon wafers,^[^ ^53^ ^]^ Liu et al. developed a novel approach for fabricating MAPbX_3_ and FAPbI_3_ thin films using diamond wire cutting (**Figure** **4**a).^[^ ^54^ ^]^ This method involved slicing bulk single crystals using a diamond wire, resulting in a 190 μm thick MAPbI_3_ SCTF with a size exceeding 50 mm (Figure 4b)^[54a]^ and a 100 μm thick FAPbI_3_ SCTF with a size over 20 mm,^[54b]^ demonstrating the scalability and large‐scale capability of this approach. Although achieving thinner SCTF through further polishing is possible, it poses a challenge due to the fragile nature of perovskite single crystal. To address this problem, Lv et al. developed a universal top‐down approach that combined primary wire‐cutting of bulk single crystals with a subsequent wet etching process in the unsaturated mother liquor.^[^ ^55^ ^]^ This method was further modified by Lv and Zhang to reduce residues on the crystal surface after the wet etching process.^[^ ^56^ ^]^ As schematically illustrated in Figure 4c, the process involved first thinning the bulk MAPbI_3_ single crystal to approximately 200 μm‐thick wafers through wire cutting. Subsequently, the crystal wafer was etched with a low‐concertation mother liquor to prevent undesired crystallization on the surface. To achieve a controlled etching rate and maintain surface uniformity, the wafer was placed on a spin‐coater and subjected to a central force to rapidly remove the solution. This method successfully produced MAPbI_3_ thin films with a thickness of less than 20 μm and a smooth surface, offering a solution to achieve SCTFs with desired thinness while preserving surface integrity.

Although slicing and wet etching provide a universal and straightforward method for preparing 3D perovskite SCTFs, they are accompanied by challenges such as surface cracks, defects, residues, and high materials waste rates, which limit their further application. In contrast, mechanical exfoliation is another top‐down method that has been adopted to produce 2D perovskite SCTFs. Liang et al. demonstrated the mechanical exfoliation of (BA)_2_(MA)_*n*‐1_PbnI_3*n*+1_ thin flakes and even the (BA)_2_(MA)_2_Pb_3_I_10_ single layer with a smooth surface.^[^ ^57^ ^]^ Similarly, other perovskite crystal flakes, such as (C_6_H_5_C_2_H_4_NH_3_)_2_PbI_4_·(CH_3_NH_3_PbI_3_)_*n*‐1_,^[^ ^58^ ^]^ (C_6_H_9_C_2_H_4_NH_3_)_2_PbI_4_,^[^ ^59^ ^]^ and (C_6_H_5_C_2_H_4_NH_3_)_2_PbBr_4_^[^ ^60^ ^]^ have also been successfully prepared via exfoliation. Although mechanical exfoliation is a straightforward and efficient method for preparing uniform and crack‐free 2D SCTFs, it is often associated with low reproducibility, and the resulting flakes are relatively small in size.

#### 2.2.2 Space‐Confined Method

**Figure 5:**
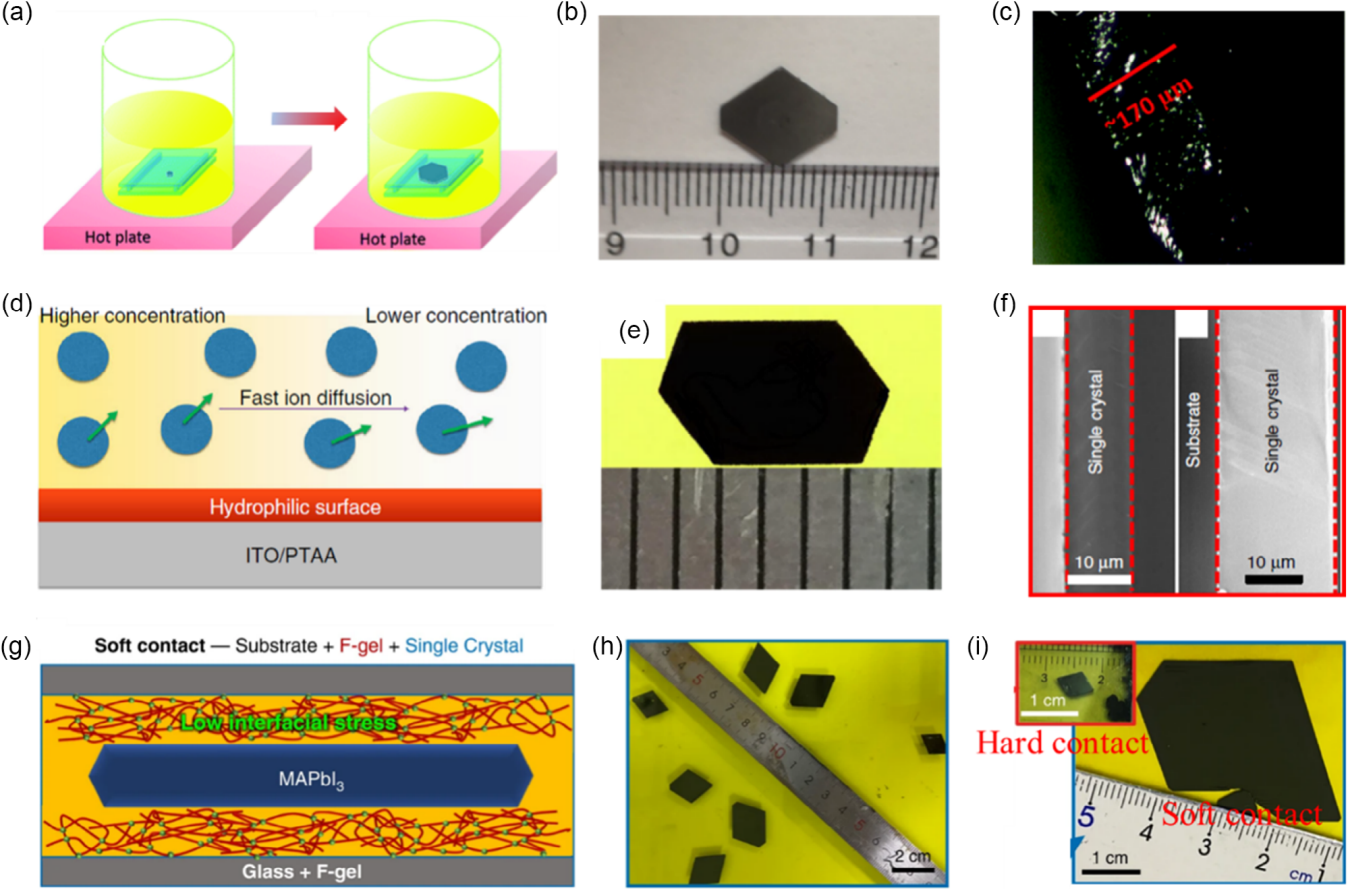
Space‐confined methods for perovskite SCTFs preparation. a) Schematic of the dynamic‐flow space‐confined method for the preparation of single crystal wafers. b) Photograph of a MAPbI_3_ single crystal wafer. c) Microscope image of MAPbI_3_ wafer with a thickness of about 170 μm. a–c) Reproduced with permission.^[^ ^61^ ^]^ Copyright 2019, the Royal Society of Chemistry. d) Growth mechanism of perovskite SCTFs on the hydrophobic substrates. e) Photograph of a MAPbI_3_ SCTF. f) Cross‐sectional SEM images of SCTFs with thicknesses of approximately 10 and 20 μm. d–f) Reproduced under the terms of the CC‐BY Creative Commons Attribution 4.0 International license (https://creativecommons.org/licenses/by/4.0).^[^ ^21^ ^]^ Copyright 2017, The Authors. Published by Springer Nature. g) Schematic of MAPbI_3_ SCTFs fabricated on the F‐gel substrate. h) Photograph of MAPbI_3_ single crystals on the F‐gel substrate. i) Enlarged photographs of the as‐fabricated MAPbI_3_ single crystals on the glass substrate (top) and soft F‐gel substrate (bottom), respectively. g–i) Reproduced under the terms of the CC‐BY Creative Commons Attribution 4.0 International license (https://creativecommons.org/licenses/by/4.0).^[^ ^68^ ^]^ Copyright 2023, The Authors. Published by Springer Nature.

As schematically depicted in **Figure** **5**a, Gao et al. used a space‐confined method to fabricate MAPbI_3_ single crystal wafers, where a seed crystal was placed between two substrates supported by spacers, allowing for precise control over the gap thickness.^[^ ^61^ ^]^ As a result, a 10 mm single crystal with a thickness of approximately 170 μm was successfully grown (Figure 5b–c). To achieve even thinner films, a pump was utilized to provide a flowing fresh solution for continuous single‐crystal growth, enabling the production of wafers with lateral sizes exceeding 1 cm and thicknesses controlled by the sandwiched spacers, reaching as low as 150 μm.^[^ ^62^ ^]^ However, despite these advancements, films with thicknesses over 100 μm still present challenges for their application in vertical devices such as solar cells and photodetectors, which typically require thinner ones. Most recently, through optimization of the pumped dynamic‐flow space‐confined system, a 35 μm thick MAPbI_3_ wafer^[^ ^63^ ^]^ and 16 μm thick MAPbBr_3_ SCTF with a size of 6 mm × 8 mm have been successfully achieved.^[^ ^64^ ^]^ Moreover, by carefully controlling the heating area, nucleation position, and growth kinetics, Li et al. have obtained high‐quality MAPbBr_3_ SCTFs with sizes exceeding 3 cm and thickness down to 15 μm.^[^ ^65^ ^]^ To achieve thinner SCTFs, Chen et al. developed a facile approach that combines space confinement with the assistance of external force to reduce the gap between two substrates.^[^ ^66^ ^]^ This method proved to be applicable for the fabrication of various perovskites SCTFs on distinct substrates. By adjusting the clamping force, the thickness of the SCTFs can be precisely controlled, ranging from tens of nanometers to several micrometers. However, it should be noted that the lateral size of these SCTFs is currently limited to hundreds of micrometers, hindering their effective application in certain devices.

Considerable efforts have been dedicated to the development of thin and large‐scale SCTFs by manipulating the perovskite nucleation barrier and reducing the nucleation density. However, when working with narrow and space‐confined gaps, the lateral growth of crystals is often constrained by the limited transport of fresh solution ions, resulting in the formation of multiple crystals or polycrystals instead of a single crystal.^[^ ^21^ ^]^ To address these challenges, Chen et al. implemented a spin‐coating process to deposit a hydrophobic hole transport layer (PTAA) on the substrate, to efficiently enhance the diffusion rate of solution ions and adjust the nucleation barrier during the growth process (Figure 5d).^[^ ^21^ ^]^ Consequently, large‐scale MAPbI_3_ and MAPbBr_3_ SCTFs with thicknesses ranging from several to tens of micrometers have been obtained (Figure 5e–f). Similarly, Yang et al. proposed a strategy to control the nucleation process and restrict the density of seed crystals by modifying the substrate surface.^[^ ^67^ ^]^ They utilized two substrates to form a space‐confined gap, with the upper silicon slice treated by a hydrophobic coating and the bottom indium tin oxide (ITO) substrate treated by oxygen plasma treatment for hydrophilic modification. As a result, they successfully achieved a MAPbBr_3_ thin film with a reduced thickness of 365 nm and a lateral size exceeding 600 μm. By tailoring the substrate surface properties, they could selectively control the nucleation and growth of the perovskite single crystals, leading to the formation of SCTFs with desired thickness and lateral dimensions. To create larger SCTFs, Song et al. modified their substrates by using a soft perfluorinated gel (F‐gel), as shown in Figure 5g.^[^ ^68^ ^]^ The F‐gel coating created a softer contact surface with lower surface energy and a larger contact angle, resulting in faster solution circulations within the confined microscale space. By utilizing this approach, researchers harvested a large number of centimeter‐sized MAPbI_3_ single crystal wafers, the largest of which measured up to 4 cm, twice the size of those grown on hard substrates (Figure 5h–i).

#### 2.2.3 Surface Tension‐Controlled Method

**Figure 6:**
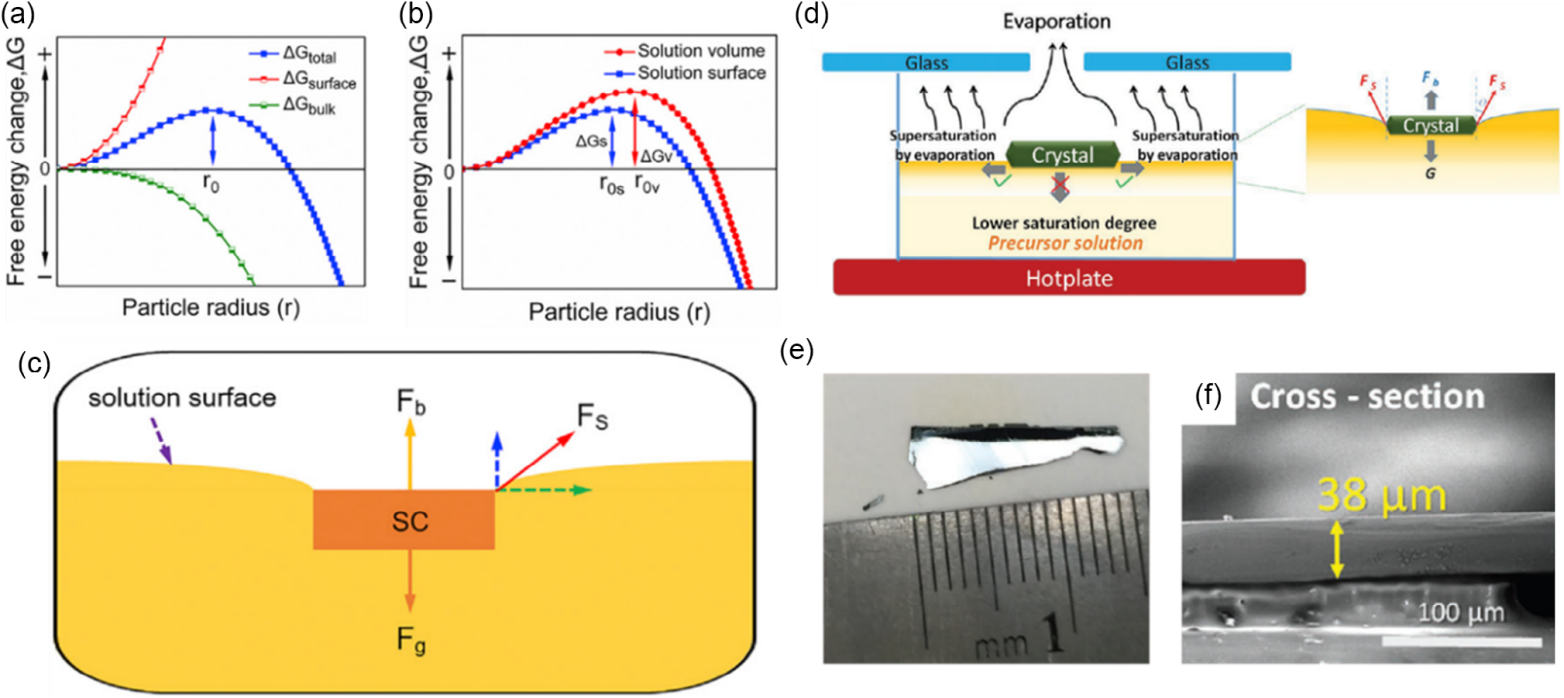
Growth mechanisms and photographs of surface tension‐controlled method. a) Schematic depicting the Gibbs free energy change of solution surface, bulk, and total. b) Graph depicting the nucleation barrier being lower at the solution surface compared to the bulk solution. c) Schematic illustration of (PEA)_2_PbI_4_ growth at air‐solution interface. a–c) Reproduced with permission.^[70b]^ Copyright 2019, Elsevier. d) Scheme of the evaporation‐controlled surface‐tension assisted MAPbI_3_ growth mechanism. e) Photograph of a MAPbI_3_ single crystal wafer with a lateral dimension of 1.5 cm. f) Cross‐sectional SEM image of MAPbI_3_ wafer. d–f) Reproduced with permission.^[^ ^71^ ^]^ Copyright 2019, John Wiley and Sons.

As previously mentioned, the exploitation of nucleation at locations with lower nucleation barriers can lead to the production of large‐scale SCTFs. Thus, Zhumekenov et al. proposed a surface tension‐controlled ITC method, which promoted lateral growth at the air‐solution interface, enabling the fabrication of free‐standing MAPbX_3_ and MASnBr_3_ SCTFs exceeding 1 cm^2^ in size.^[^ ^69^ ^]^ This method has not only been limited to 3D perovskites but has also been successfully applied to fabricate 2D perovskite large‐scale SCTFs.^[^ ^70^ ^]^ As schematically illustrated in **Figure** **6**a, the nucleation process occurs when the solution becomes oversaturated, and owing to the surface tension effect, the surface nucleation barrier is lower than that of the bulk solution, resulting in a higher probability of nucleation at the solution surface (Figure 6b). Additionally, the surface tension force allows the small crystals to be suspended on the solution surface, promoting faster growth of the lateral edges compared to the vertical direction of the crystal (Figure 6c). Finally, inch‐sized 2D (PEA)_2_PbI_4_ SCTFs up to 36 mm in length were obtained through careful control of the solution cooling rate (0.5 °C h^−1^ from 105 °C to 30 °C).^[70b]^

Other 2D perovskites, such as (BA)_2_(MA)_*n*−1_Pb_*n*_I_3*n*+1_ with varying layers^[70a]^ and BA_2_PbBr_4_, can be fabricated with thicknesses as thin as tens of nanometers and lateral sizes exceeding 6 cm,^[70d]^ highlighting the versatility of this approach for various perovskites. To expedite the preparation of large‐scale single‐crystal wafers using the surface tension‐controlled method, Liu et al. introduced an inorganic HI aqueous solution as the medium.^[^ ^71^ ^]^ As shown in Figure 6d, MAPbI_3_ nucleation was controlled through water solvent evaporation, leading to precursor solution supersaturation and faster growth in the lateral direction driven by surface tension. Within just 30 min, a high‐quality MAPbI_3_ SCTF measuring 1.5 cm in length with a smooth surface and a thickness of 38 μm was obtained (Figure 6e–f). However, the surface tension‐controlled method is limited to the growth of freestanding SCTFs, and the use of solvents with a high surface tension coefficient is preferred, which may impose certain limitations on its broader applications.

#### 2.2.4 Chemical Vapor Deposition Method

As a prominent member of the family of vapor‐phase growth techniques (sputtering, evaporation, molecular‐beam epitaxial, etc.), the CVD method is a well‐established approach for the controlled kinetic growth of single crystals. Generally, this method relies on the transportation of precursor molecules through a carrier gas to a target substrate maintained at a specific temperature. Upon reaching the substrate, the precursors undergo chemical reactions and subsequently grow into various morphologies, influenced by the properties of the substrates and materials involved. Epitaxial growth occurs when a single crystal substrate with a well‐matched crystal lattice to the material enables the growing film to mimic the substrate's crystal structure, leading to high‐quality SCTFs with excellent structural alignment. Through careful optimization of the growth conditions (temperature, pressure, flux rate, time, etc.), researchers can successfully obtain SCTFs with desired thicknesses and crystallographic orientations. The versatility and controllability of the CVD technique make it a valuable tool for the synthesis of high‐quality IV, III–V, and II–VI semiconductor SCTFs and heterostructures,^[^ ^72^ ^]^ and has also been recently extended to the preparation of perovskite SCTFs.

**Figure 7:**
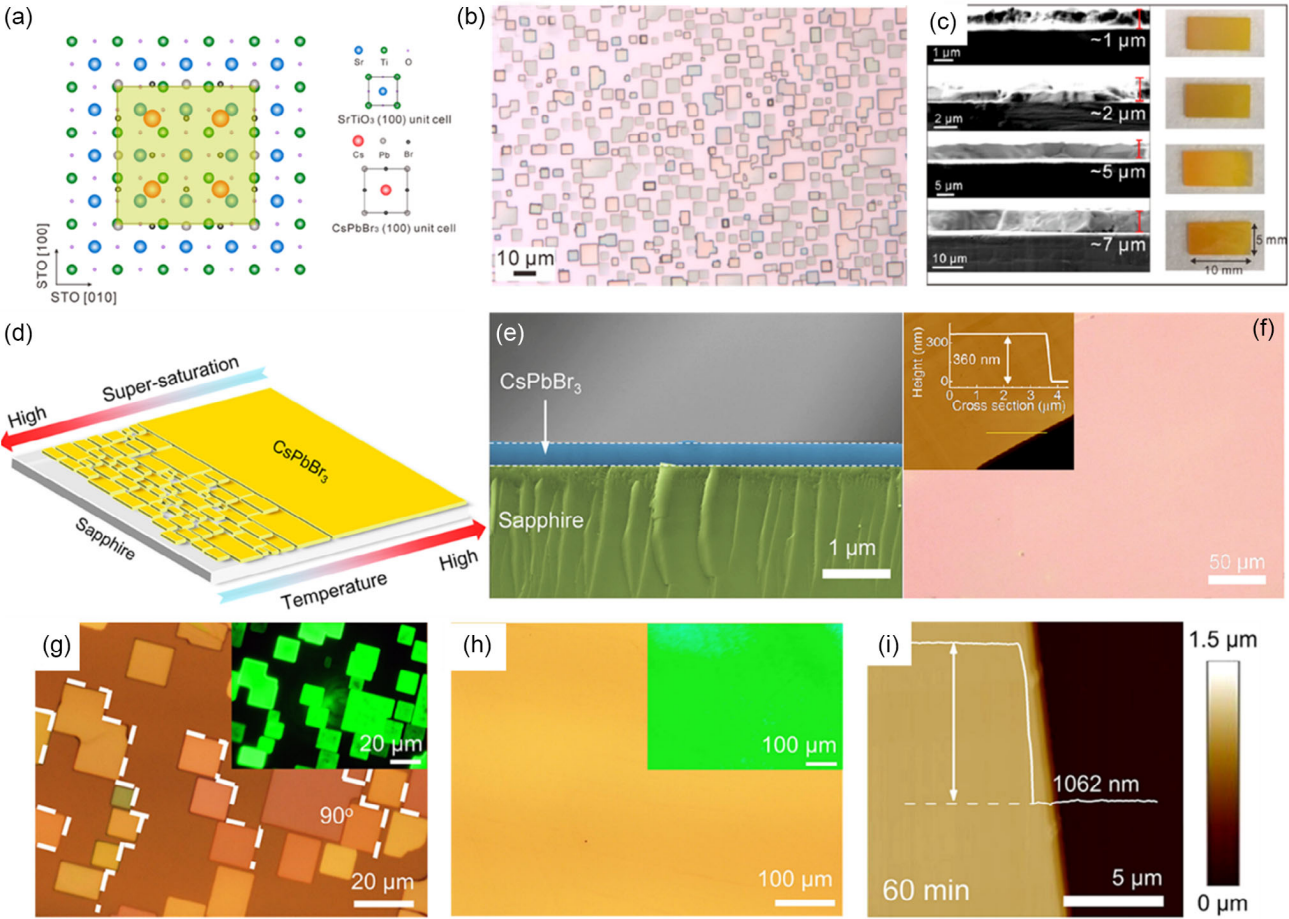
Growth mechanisms and photographs of the CVD method. a) Schematic of incommensurate lattice matching of the (100) planes between the cubic‐phase CsPbBr_3_ and the STO substrate. b) Optical image of CsPbBr_3_ nano/microplates epitaxially grown on STO substrates. c) Cross‐sectional SEM images and optical images of the as‐fabricated CsPbBr_3_ thin films with different thicknesses. a–c) Reproduced with permission.^[^ ^74^ ^]^ Copyright 2017, the American Chemical Society. d) Schematic illustration of the nucleation and merge process of CsPbBr_3_ thin film prepared on *c*‐plane sapphire (001) substrates. e,f) Cross‐sectional SEM and AFM images of CsPbBr_3_ thin film on *c*‐plane sapphire (001) substrates, respectively. d–f) Reproduced with permission.^[^ ^75^ ^]^ Copyright 2020, the American Chemical Society. g) The optical image of separately aligned CsPbBr_3_ microplates, while the inset is the corresponding photoluminescence (PL) image. h) The optical image of merged CsPbBr_3_ thin film with a smooth, pinhole‐free surface, while the inset is the corresponding PL image. i) AFM image of the edge of merged CsPbBr_3_ thin film. g–i) Reproduced with permission.^[^ ^78^ ^]^ Copyright 2021, the American Chemical Society.

Wang et al. successfully fabricated all‐inorganic perovskite SCTFs on NaCl single crystal substrates through epitaxy growth, capitalizing on their similar material chemistries and crystal lattices.^[^ ^73^ ^]^ The resulting centimeter‐sized CsPbBr_3_ and CsSnBr_3_ SCTFs exhibited smooth surfaces and tunable thicknesses ranging from 200 nm to 7 μm, depending on the growth time.^[^ ^73^ ^]^ Chen et al. utilized the CVD method to prepare CsPbBr_3_ SCTFs on (100)‐oriented SrTiO_3_ (STO) substrates.^[^ ^74^ ^]^ Despite the lattice mismatch between CsPbBr_3_ and the STO substrate, the close arrangement of two unit cells of CsPbBr_3_ with three unit cells of STO allowed for favorable epitaxial growth, as shown in **Figure** **7**a. During the high temperature (≈450 °C) heteroepitaxial growth process, nano/microplates undergo gradual formation and ultimately coalesce into continuous SCTFs (Figure 7b). By controlling the growth time, the thickness of thin films can be tuned within the range of 1 to 7 μm, while the overall film size is determined by the dimensions of the STO substrates (Figure 7c). Recently, Zhang's group demonstrated the deposition of CsPbBr_3_ microstructures and large‐scale SCTFs on various substrates, such as sapphire,^[^ ^75^ ^]^ GaN/sapphire,^[^ ^76^ ^]^ and SiO_2_/Si^[^ ^77^ ^]^ substrates. By using *c*‐plane sapphire as the substrate, they identified the crucial role of high temperature in fabricating continuous thin SCTFs, as it facilitated atomic diffusion, increased the diffusion length, controlled the reactant concentration, and promoted the growth of merged films (Figure 7d).^[^ ^75^ ^]^ Consequently, a CsPbBr_3_ SCTF sized 330 μm × 250 μm with a thickness of 360 nm was achieved, which exhibited an ultra‐smooth surface with an estimated roughness of 2.1 nm (Figure 7e,f). Furthermore, Wang et al. fabricated a centimeter‐sized CsPbBr_3_ thin film on muscovite mica, where the epitaxial alignment of single crystal microplates on the substrate was facilitated by their matched lattice constants (Figure 7g).^[^ ^78^ ^]^ The microplates continued to grow and merge, resulting in a smooth thin film measuring 2 cm in size, with a controlled thickness of just over 1 μm, showing a high lateral size‐to‐thickness ratio of 2 × 10^4^ (Figure 7h–i). Other studies have also highlighted the potential of the CVD method in producing large‐scale CsPbBr_3_ SCTFs,^[^ ^79^ ^]^ further emphasizing the capability of the CVD method for fabricating high‐quality all‐inorganic perovskite SCTFs and enabling the exploration of high‐performance optoelectronic applications.

Lastly, we would like to discuss some strategies for obtaining large‐scale perovskite single crystals while maintaining their high quality, which may provide valuable insights for further exploration in this field. On one hand, for growing large‐scale and high‐quality bulk single crystals, solution methods, particularly the ITC method, have proven to be the most popular approach. By repeatedly supplying the perovskite solution with fresh precursor or continuing feeding with fresh precursor solutions, the crystal can be supplied with an ample amount of raw material, allowing for the growth of a single crystal exceeding two inches in size.^[^ ^24^, ^62^, ^80^ ^]^ Meanwhile, to reduce the defect density of the crystals, additives can be incorporated into the precursor solutions to regulate the nucleation and growth processes.[^14^, ^81^] For instance, Ma et al. reported a polymer‐controlled route that effectively reduced the nuclei concentration and resulted in the growth of centimeter‐sized perovskite single crystals with high quality.^[^ ^82^ ^]^ Moreover, lowering the reaction temperature can also help minimize defect density caused by high temperatures. Alsalloum et al. utilized a mixture solvent of propylene carbonate (PC) with GBL to reduce the crystallization temperature from 130 °C to 90 °C, thereby reducing the escape of MAI from the crystal surface and achieving high‐quality MAPbI_3_ single crystals.^[^ ^83^ ^]^ Similarly, Cho et al. increased the solubility of MAPbBr_3_ by adding dry ice and successfully grew sizeable high‐quality MAPbBr_3_ single crystals at low temperatures.^[^ ^84^ ^]^

On the other hand, for fabricating large‐scale perovskite SCTFs, the top‐down strategy developed by Liu et al. and the CVD method developed by Zhong et al. have emerged as two of the most efficient approaches.^[^ ^54^, ^75^ ^]^ However, the limitations of free‐standing structures, thick films, and substrate restrictions may restrict their widespread applications in high‐performance integrated devices. To overcome these challenges, substrates treatment such as the use of PTAA to enhance ion transport in micrometer‐sized gaps are widely accepted. Chen et al. pioneered the use of this strategy to prepare high‐quality MAPbI_3_ SCTFs, achieving films with thicknesses as low as 10 μm.^[^ ^21^ ^]^ Similarly, Deng et al. successfully grew high‐quality MAPbBr_3_ SCTFs with trap densities down to 10^11^ cm^−3^ and mobility over 60 cm^2^ V^−1^ s using the space‐confined method with hydrophobic substrates. The resulting SCTFs exhibited lateral sizes exceeding 1 cm and thicknesses down to 10 μm. These approaches and techniques are increasingly becoming the primary methods for preparing large‐scale and high‐quality single crystals.

## 3 Patterning on Single Crystal Perovskite Surfaces

In addition to the efforts focused on improving the intrinsic photoelectric properties of perovskites, such as the aforementioned approaches for the preparation of high‐quality bulk crystals and SCTFs, the development of patterning technologies has emerged as a crucial tool for fabricating nano‐ or micropatterns in optoelectronic devices using perovskites in various applications. Numerous studies have shown that constructing functional patterns on the surfaces or within the active layers can enhance or control the optical and electrical properties.^[^ ^85^ ^]^ One of the commonly used approaches for patterning polycrystalline perovskite thin films is photolithography, which is a well‐established method in the semiconductor industry. Subsequently, various techniques have been employed to fabricate patterned perovskite films, including imprinting,^[^ ^86^ ^]^ micromolding in capillaries,^[^ ^87^ ^]^ inkjet printing,^[^ ^88^ ^]^ and microcontact printing,^[^ ^89^ ^]^ and so forth. These advances in patterning polycrystalline thin films have demonstrated their attractive properties in many fields and have been previously summarized elsewhere.[^38^, ^90^] However, the aforementioned methods are not suitable for patterning specific structures on single‐crystal surfaces, limiting the combining of unique patterning characteristics with the excellent properties of single crystals. Fortunately, a variety of advanced top‐down (focused ion beam (FIB), electron‐beam lithography (EBL), and laser irradiation) and bottom‐up (epitaxial growth) methods have been adopted to design and fabricate specific patterns on single‐crystal surfaces, showcasing their exceptional optoelectronic properties. This section provides a summary of popular techniques for patterning on single‐crystal surfaces.

### 3.1 Top‐Down Fabrication

**Figure 8:**
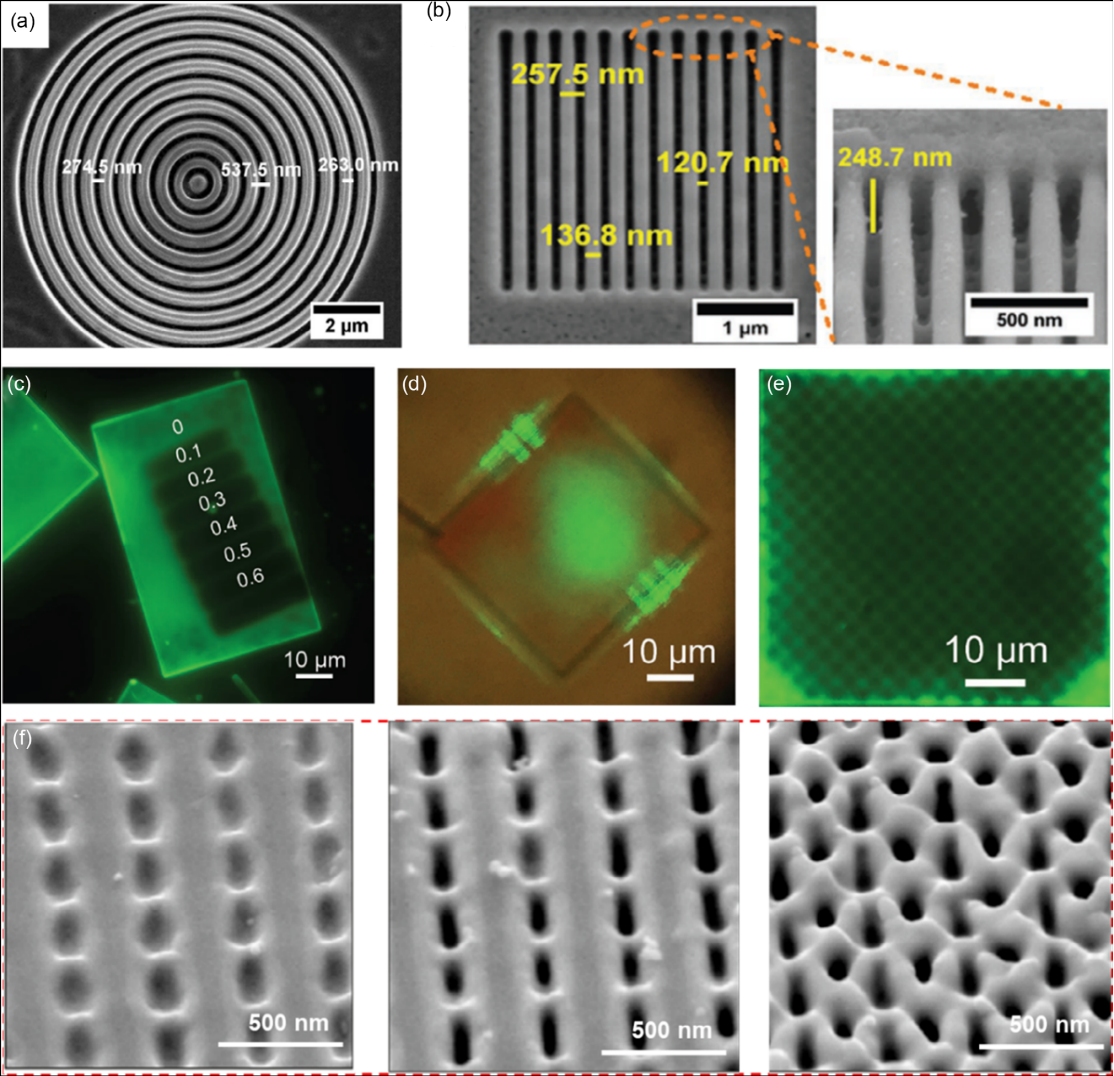
Various perovskite single‐crystal surface patterns fabricated by different top‐down technologies. a) SEM image of circular MAPbBr_3_ patterns fabricated using Ga^+^ ions FIB. Reproduced under the terms of the CC‐BY Creative Commons Attribution 4.0 International license (https://creativecommons.org/licenses/by/4.0).^[^ ^91^ ^]^ Copyright 2015, The Authors. Published by the American Vacuum Society. b) SEM image of line patterns on MAPbBr_3_ surface designed using gas‐assisted FIB. Reproduced with permission.^[^ ^92^ ^]^ Copyright 2016, the American Chemical Society. c) PL image of MAPbBr_3_ microplate treated using different e‐beam doses. d) PL image of a 1D grating MAPbBr_3_ microplate patterned by e‐beam irradiation. e) PL image of MAPbBr_3_ microplate patterned with surface circles by EBL. c–e) Reproduced with permission.^[97a]^ Copyright 2017, John Wiley and Sons. f) SEM images of CsPbBr_3_ surface morphology with elevated pulse energy. Reproduced with permission.^[101a]^ Copyright 2021, John Wiley and Sons.

In the early stages, a FIB was employed to etch perovskite single crystals directly, enabling the creation of surface patterns. In 2015, Alias et al. utilized FIB with a 17 nm diameter Ca^+^ source to etch the exposed surface of bulk MAPbBr_3_ crystals, resulting in circular grating patterns with a period of approximately 540 nm (**Figure** **8**a).^[^ ^91^ ^]^ The same group later improved the etching process by using gas‐assisted FIB with XeF_2_ and I_2_ vapors, resulting in surface damage recovery, enhanced etching, and better PL properties, and reduced pattern size down to sub‐micrometer periodic lines with a domain size of 137 nm (Figure 8b).^[^ ^92^ ^]^ FIB‐etched patterns have also been applied to the surface of CsPbBr_3_ microplates^[^ ^93^ ^]^ and nanowires,^[^ ^94^ ^]^ where the Ga^+^ ion beam facilitated selective area etching transfer to the defected regions.^[^ ^93^ ^]^

Although direct patterning using FIB offers advantages such as mask‐free and alignment‐free operation, the high‐energy ions used can potentially induce damage to the perovskites, leading to the formation of defective or amorphous phases even at low ion doses. Alternatively, EBL could be a nondestructive or less damaging method for patterning single‐crystal surfaces in certain cases. The e‐beam can directly create patterns on a resist layer, typically using Poly(methyl methacrylate) (PMMA) as the commonly used resist material. PMMA can be spin‐coated onto the surface of the single crystal, and then exposed to the e‐beam to generate desired patterns based on the designed structures. The exposed area of the PMMA can be easily removed by immersion in solvents without damaging the underlying perovskite. These patterned PMMA layers then serve as masks for transferring the patterns onto the perovskite surface, followed by the complete removal of the remaining PMMA.^[^ ^95^ ^]^ Song's group^[^ ^96^ ^]^ demonstrated that using EBL patterning and subsequent etching processes, designed shapes such as circular disks, rectangles, and polygons of perovskite single‐crystal microplates can be fabricated, exhibiting superior optical properties. Interestingly, direct exposure of perovskite single crystals to e‐beams can lead to material destruction and decomposition, which can be exploited to tailor the device's performance.^[^ ^97^ ^]^ For instance, e‐beam‐exposed areas showed reduced PL, allowing for the design of line patterns on MAPbBr_3_ microplates (Figure 8c–d). Unpatterned lines on the microplate exhibited intense PL and even demonstrated lasing emission.^[97a]^ Circle patterns were also achieved by direct exposure to the single‐crystal surface (Figure 8e).

While EBL is a useful tool for preparing large‐area patterns, its widespread application is hindered by challenges such as imprecise control of dose and power, as well as the long‐time‐consuming scanning of the e‐beam. Laser writing, in contrast, offers a promising alternative to EBL and FIB. By utilizing a high‐energy laser, laser writing can selectively heat or remove specific regions of materials through the laser ablation effect, providing a more efficient and economical method for patterning. In 2017, Steele et al. demonstrated the use of a 458 nm continues‐wave laser to locally heat the FAPbI_3_ single‐crystal surface, leading to the transformation of the nonperovskite δ‐phase FAPbI_3_ into the perovskite α‐phase FAPbI_3_ with microscale line gratings. These regions exhibited highly luminescent and long‐term phase stability.^[^ ^98^ ^]^ Laser writing provides precise control over power, frequency, time, and location temperature, making it an effective tool for surface patterning. Xing et al. utilized femtosecond laser ablation to create microstructures on the surface of MAPbBr_3_ single crystals, resulting in a two‐order enhancement of PL intensity.^[^ ^99^ ^]^ Subsequently, laser writing has been employed to directly pattern various single crystal perovskites, such as FAPb(Br_*x*_I_1‐*x*_)_3_,^[^ ^100^ ^]^ CsPbBr_3_,^[^ ^101^ ^]^ and quasi‐2D (BA)_2_(MA)_*n*‐1_Pb_*n*_I_3*n*+1_.^[^ ^102^ ^]^ Figure 8f illustrates the capability of laser ablation to create high spatial resolution nanostructures, where the size and depth of the patterns increase with higher pulse energy from left to right.^[101a]^ Note that the laser ablation process leaves behind clean surfaces after patterning. In addition, laser writing shows several other advantages, including mask‐free patterning, precise control over positions, and reduced processing time, attributing it as a promising approach for various applications in patterned devices.

### 3.2 Bottom‐Up Growth

**Figure 9:**
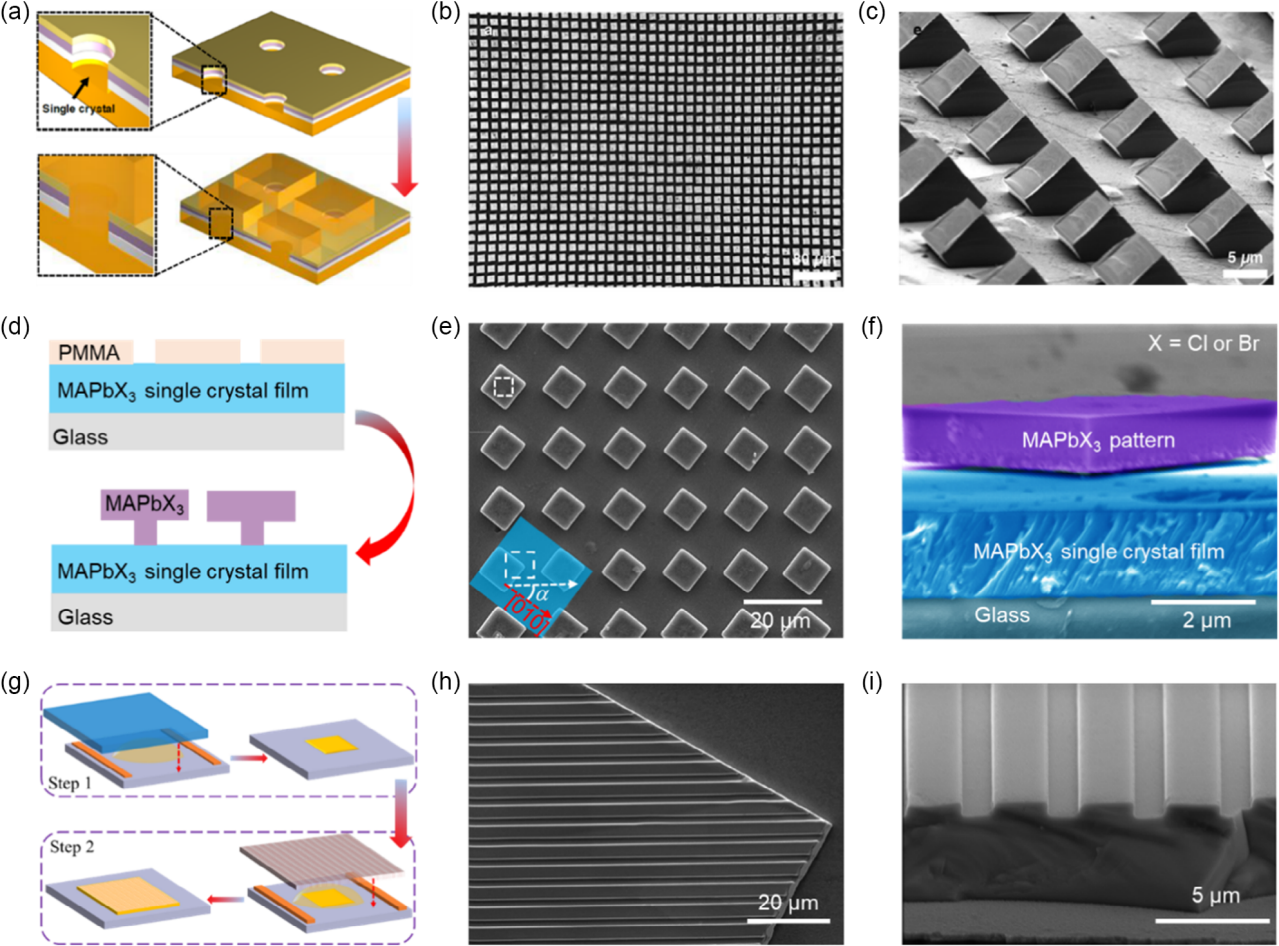
Various perovskite single‐crystal surface patterns fabricated by different bottom‐up technologies. a) Schematic of etching protective layers and the growth process patterns. b) Top‐view SEM image of epitaxial MAPbBr_3_ arrays. c) Tilted‐view SEM image of crystal orientation control direction. a–c) Reproduced with permission.^[^ ^103^ ^]^ Copyright 2018, John Wiley and Sons. d) Schematic of PMMA protective layer etching and MAPbX_3_ patterns. e) Top‐view SEM image of suspended MAPbBr_3_ microarrays. f) Cross‐sectional SEM image of MAPbX_3_ SCTF with suspended microplate on the surface. d–f) Reproduced with permission.^[^ ^109^ ^]^ Copyright 2022, John Wiley and Sons. g) Schematic of the two‐step method to fabricate surface patterned MAPbBr_3_ SCTF. h) Top‐view and i) cross‐sectional SEM images of surface patterned MAPbBr_3_ thin film, respectively. g–i) Reproduced with permission.^[^ ^110^ ^]^ Copyright 2020, the American Chemical Society.

Epitaxial growth, encompassing homo‐ and heterointerface, on lattice‐constant matched substrates, has emerged as a conventional method for designing semiconductor patterns, which has garnered considerable attention due to its capability to fabricate perovskite single crystal patterns with reduced interfacial defect density. In a recent study by Xu's group, homoepitaxial growth of microcrystal arrays on the surface of MAPbBr_3_ bulk single crystal was demonstrated (**Figure** **9**a).^[^ ^103^ ^]^ Mask layers were deposited onto the single crystal, creating micrometer‐sized holes through etching processes. Subsequent epitaxial growth was achieved by immersing the sample into MAPbBr_3_ DMF‐saturated precursor solutions, with various morphologies, and crystal orientations achieved through precise control of substrate crystal planes, growth conditions, and mask geometry (Figure 9b,c).^[^ ^103^ ^]^ Using a similar method, the same group also successfully demonstrated large‐area homoepitaxial growth of MAPbI_3_ single‐crystal surface patterns^[^ ^104^ ^]^ and heteroepitaxial growth of 2D BA_2_SnI_4_ superlattice patterns on a MAPb_0.5_Sn_0.5_Br_3_ substrate.^[^ ^105^ ^]^ These surface patterns could merge into high‐quality centimeter‐sized SCTFs for further optoelectronic studies. Cui et al. also reported the successful utilization of mask‐assistant epitaxial growth to fabricate MAPbBr_3_ pattern arrays on an oriented CsPbBr_3_ substrate. By optimizing the solution growth zones, they showcased the versatility of this method for achieving single crystal pattern growth. However, the challenge of mask removal after epitaxial growth has limited its applications in certain areas such as suspended microdevices, which have shown advancements in microlasers,^[^ ^106^ ^]^ memories,^[^ ^107^ ^]^ sensors,^[^ ^108^ ^]^ and other fields. To overcome this obstacle, Zhang et al. proposed a novel approach for fabricating suspended single‐crystal microarrays, as schematically shown in Figure 9d.^[^ ^109^ ^]^ In their process, a protective PMMA was spin‐coated onto the MAPbX_3_ SCTF and exposed to an e‐beam to form designed patterns. The exposed areas could be selectively removed using a mixture of solvents that were specifically passivated to the underlying perovskite, ensuring the integrity and protection of the perovskite material. The thin film with a PMMA mask was immersed into an HX‐based MAPbX_3_‐saturated aqueous solution, and after the epitaxial process, the PMMA layer was removed using chlorobenzene and hexanes, resulting in clean and smooth suspended MAPbX_3_ arrays (Figure 9e). The gap between the substrate and array could be observed clearly (Figure 9f). This simple and nondestructive method holds promise for applications in suspended devices.

To simplify the preparation of the single‐crystal surface patterns, Zhang et al. proposed another simple and effective method, as schematically shown in Figure 9g.^[^ ^110^ ^]^ In the first step, a MAPbBr_3_ SCTF was prepared using a space‐confined strategy, serving as a seed layer. In the second step, the upper substrate was replaced with PDMS containing predesigned nano‐/microstructures. With further growth of the film in a saturated MAPbBr_3_ solution, the morphology of PDMS was transferred to the surface of MAPbBr_3_ SCTF. This two‐step surface pattern method resulted in a clean and smooth surface grating with a period from 4.5 μm (Figure 9h) to 740 nm (depth of 80 nm). Meanwhile, the film size reached up to 5 mm with a thickness of approximately 5 μm (Figure 9i). This method provides flexibility for creating arbitrary structures by changing the PDMS patterns.

Following similar structure‐transfer principles, Zhang et al.^[^ ^111^ ^]^ and Li et al.^[^ ^112^ ^]^ demonstrated the one‐step preparation of MAPbBr_3_ microplates using imprinted PDMS and polyethylene as templates, respectively. In addition to the template transfer onto the single‐crystal surface, the deposited solution on a single crystal can self‐assemble to form random pattern arrays, such as the 2D on 3D ((PEA)_2_PbBr_4_/CsPbBr_3_)^[^ ^113^ ^]^ heterostructures and the 3D on 2D (CsPbBr_3_/PEA_2_PbBr_4_, CsPb(Br_*x*_Cl_1‐*x*_)_3_/PEA_2_Pb(Br_1‐*y*_Cl_*y*_)_4_, etc.)^[^ ^114^ ^]^ heterostructures. These achievements open opportunities for investigating distinct carrier transfer phenomena in different heterojunctions. In conclusion, epitaxial growth provides a promising method for growing defect‐free interfacial junctions and heterostructures, serving as platforms for studying the phenomena arising from lattice strain.

For comparison, we summarize the advantages and disadvantages of the methods mentioned above, as shown in **Table** **1**. In terms of bulk single‐crystal preparation, all the above‐mentioned methods offer the advantage of growing large‐scale and high‐quality crystals. The ITC method stands out as the most efficient among them, while the Bridgeman method can yield high‐quality crystals with ultralow defect density, yet requires high‐purity raw material and high‐temperature conditions. In contrast, SCTFs can be prepared using various approaches. Wafer slicing is one of the direct top‐down methods capable of designing large‐scale and high‐quality films. However, due to the fragile nature of perovskites, the thickness of the final wafer is limited, and wet etching can be employed to further reduce the thickness based on the sliced wafers. The space‐confined method is an effective approach for growing high‐quality SCTFs with controllable thickness; however, careful attention must be given to addressing the high‐density defects on the surfaces. Another timesaving method is the surface tension assistant method, which is suitable for producing large‐scale SCTFs yet limited to free‐standing wafers. The CVD method has been employed to grow lattice‐matched SCTFs on target substrates, enabling the achievement of large size‐to‐thickness ratio. Additionally, top‐down patterning techniques such as FIB, EBL, and laser ablation offer the advantages of mask‐free and position‐controllable patterning; however, the high‐energy ions or photons may have a devastating effect on the crystal surface. Alternatively, epitaxial growth from solution can circumvent these drawbacks, yet in certain cases still requires mask assistance and involves complex processes.

**Table 1:** Comparison of the advantages and disadvantages of the methods

Method	Advantages	Disadvantages
ITC	Large scale; high quality; timesaving	Sensitive to impurities
LTC	Large scale; high quality	Time‐consuming
AVC	Large scale; high quality	Time‐consuming
Bridgeman	Large scale; high quality	High‐purity raw material; high temperature
Wafer slicing	Large scale; high quality	Too thick; only limited to free‐standing wafer
Wafer slicing + Wet etching	Thickness controllable to several micrometers	Complex processes; only limited to free‐standing film
Space confinement	Thickness‐controllable; high quality	High defect density on the surface
Surface tension assistant	Time‐saving; large scale	Too thick; only limited to free‐standing film
CVD	Large scale; thickness‐controllable	Substrate selectivity; high temperature
FIB	Mask‐free; position‐controllable	Destructive; size‐limited
EBL	Mask‐free; position‐controllable	Destructive; size‐limited
Laser ablation	Mask‐free; position‐controllable	Destructive; size‐limited
Epitaxial growth	Nondestructive; orientation‐controllable	Mask‐assistant; complex processes

## 4 Optoelectronic Applications

Single crystals with fewer defects, traps, and grain boundaries are highly desired for optoelectronic applications. Perovskite single crystals, in particular, demonstrate outstanding carrier transport characteristics with high mobility of charge carriers (hundreds of cm^2^ V^−1^ s^−1^), relatively long carrier lifetimes (several microseconds), and long carrier diffusion lengths (over 100 μm).[^4^, ^115^] Furthermore, perovskites showcase tuneable exciton binding energies (*E*_b_) depending on their compositions and dimensions. For instance, MAPbI_3_ exhibits an *E*_b_ of approximately 10 meV at room temperature, significantly lower than the thermal energy of around 26 meV,^[^ ^116^ ^]^ suggesting that excitons can readily convert into free charge carriers by surmounting the Coulombic interaction fluctuations.^[^ ^117^ ^]^ Remarkably, by reducing its dimensions or altering its composition, the *E*_b_ can be increased to several hundred multielectron volts.^[^ ^118^ ^]^ This flexible adjustment ability of *E*_b_ renders perovskite highly versatile for applications in variable photovoltaics/photodetectors (with small *E*_b_) and light‐emitting devices (with large *E*_b_).

The utilization of low‐temperature and facial solution‐processed methods, along with various crystallization approaches, has resulted in the rapid and widespread adoption of perovskite single crystals in diverse application explorations. Meanwhile, functional patterning techniques are essential for flexible and intelligent optoelectronics and light‐emitting applications to facilitate the creation of novel and distinctive surface patterns on perovskite single crystals. Consequently, discussing and evaluating the performances of optoelectronic devices based on perovskite single crystals is crucial in comprehending the challenges associated with materials preparation and device fabrication.

In this section, we aim to showcase the remarkable progress made in the development of perovskite single‐crystal devices, with a specific focus on their diverse morphologies, encompassing bulk single crystals, SCTFs, and single crystals with surface patterns. Besides, we also emphasize the practical applications of these devices, including photodetectors/irradiation photodetectors, solar cells, lasers, and so forth.

### 4.1 Bulk Single Crystal Devices

The thickness of bulk crystals plays a crucial role in determining the performance of devices based on them. To accommodate different thickness requirements, photodetectors can be fabricated using planar‐type and vertical‐type structures. For instance, as for irradiation photodetection, a vertical‐type perovskite detector with a large thickness is necessary to ensure the complete absorption of high‐energy photons. Hence, bulk single crystals are extensively studied for achieving high performance in photodetectors.

**Figure 10:**
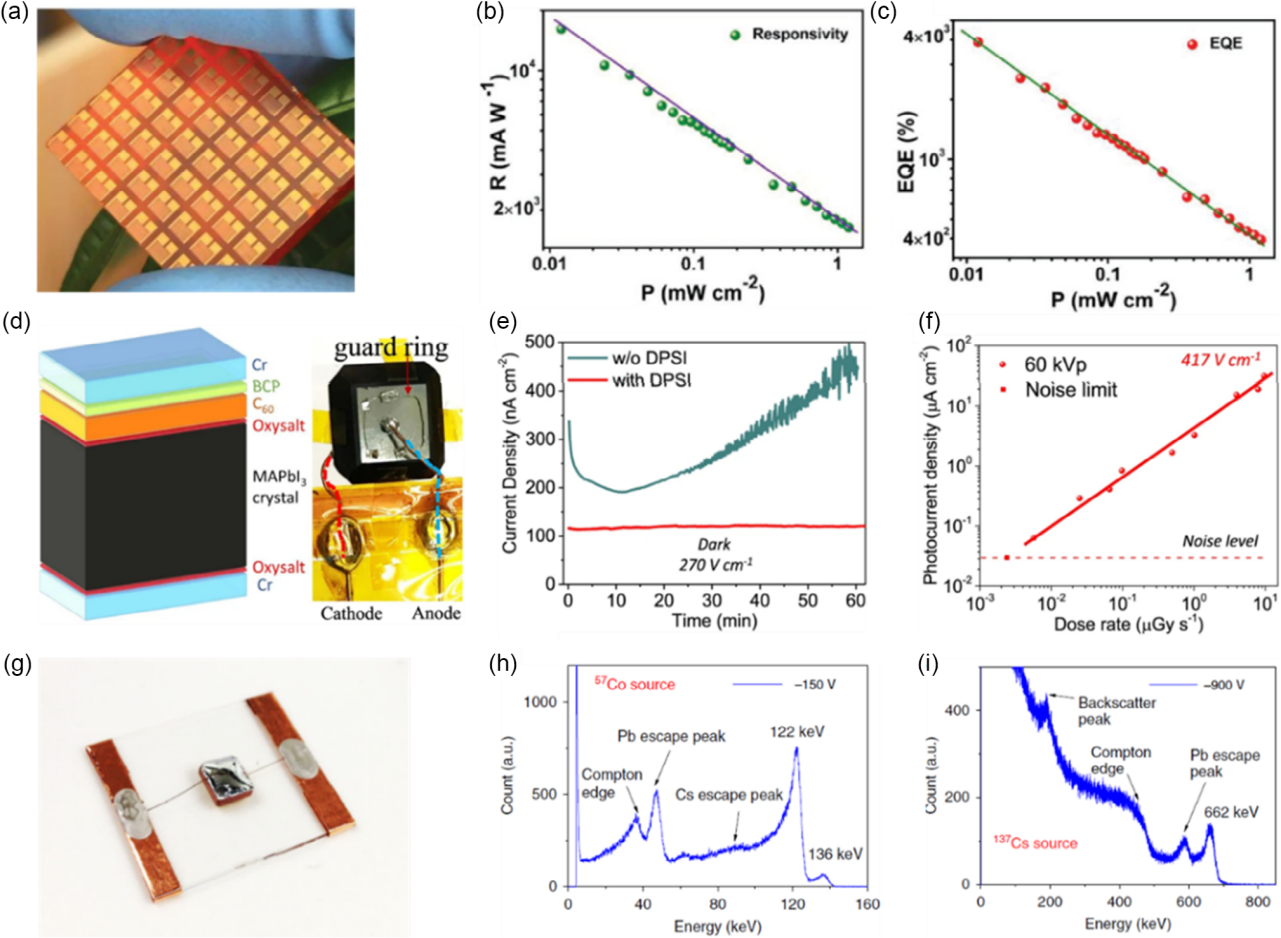
Photodetectors based on perovskite bulk single crystals. a) Photodetector arrays fabricated on MAPbBr_3_ single crystal. b) Power‐dependent responsivity (R) of MAPbBr_3_ detector. c) Power‐dependent EQE of MAPbBr_3_ detector. a–c) Reproduced with permission.^[^ ^120^ ^]^ Copyright 2018, John Wiley and Sons. d) Schematic of the X‐ray detector made of MAPbI_3_ single crystal and a photo of the device. e) Dark current density measured in MAPbI_3_ devices with and without DPSI. f) Output current of the MAPbI_3_ device with DPSI under various dose rates. d–f) Reproduced under the terms of the CC‐BY Creative Commons Attribution 4.0 International license (https://creativecommons.org/licenses/by/4.0).^[14b]^ Copyright 2021, The Authors. Published by Springer Nature. g) Photo of a typical Ga/CsPbBr_3_/Au detector. h,i) Energy resolved spectrum of ^57^Co γ‐ray and ^137^Cs γ‐ray sources, respectively. g–i) Reproduced under the terms of the CC‐BY Creative Commons Attribution 4.0 International license (https://creativecommons.org/licenses/by/4.0).^[50c]^ Copyright 2018, The Authors. Published by Springer Nature.

Planar photodetectors, consisting of two electrodes on the same device surface, have gained significant attention due to their ease of fabrication. In 2015, Lian et al. reported the successful development of a planar photodetector using the (100) surface of MAPbI_3_ single crystal, demonstrating superior performance compared to its polycrystalline thin film counterpart. It demonstrated a 100‐fold increase in both responsivity and external quantum efficiency (EQE), along with a 1,000‐fold faster response speed, attributed to lower trap densities and longer carrier diffusion lengths.^[^ ^36^ ^]^ Moreover, high‐quality MAPbCl_3_ bulk single crystals have been demonstrated as highly effective ultraviolet photodetectors, exhibiting a responsivity of 3.73 A W^−1^ (1 mW cm^−2^) and an ultrafast rise time of 130 ns.^[^ ^119^ ^]^ Furthermore, Liu's group has successfully fabricated photodetector arrays on the surface of bulk perovskite single crystals, enabling the production of imaging devices with individual sensor pixels and compatibility with large‐scale integrated circuits.^[^ ^81^, ^120^ ^]^ As depicted in **Figure** **10**a, 7 × 8 photodetector arrays were produced on MAPbBr_3_ single crystals, demonstrating high calculated responsivity, EQE, and detectivity values of 1.6 × 10^4^ mA W^−1^ (Figure 10b), 3900% (Figure 10c), and 6 × 10^13^ Jones, respectively.^[^ ^120^ ^]^ Moreover, lead‐free inch‐sized bulk (TMHD)BiBr_5_ exhibited a promising on/off ratio (≈10^3^) and a fast response speed (≈10 ms), highlighting its potential for nontoxicity perovskite optoelectronic applications.^[^ ^121^ ^]^ In addition to (TMHD)BiBr_5_, inch‐scale planar photodetectors using low‐dimensional (BA)_2_PbI_4_ have also been successfully fabricated, showcasing low defect density, uniform surface, and long‐term stability, and excellent performance with an extremely low dark current (≈10^−13^ A) and a higher on/off ratio (up to 10^4^).^[70a]^

In the field of vertical‐type photodetectors utilizing bulk single crystals, high‐energy photodetectors have been extensively researched. In 2016, Huang et al. reported the development of a sensitive X‐ray photodetector using MAPbBr_3_ bulk crystal.^[^ ^122^ ^]^ To improve carrier transport length and suppress undesired dark current, they employed a nonstoichiometric ratio of 0.8 (PbBr_2_/MABr) for high‐quality crystal growth, resulting in an ultra‐high mobility‐lifetime product of up to 1.2 × 10^−2^ cm^2^ V^−1^. Additionally, the surface defects of MAPbBr_3_ were effectively passivated through UV‐O_3_ treatment, which improved carrier lifetime and efficiency by reducing surface charge recombination velocity. The resulting device exhibited exceptional sensitivity of 80 μC Gy_air_ cm^−2^, surpassing that of α‐Se X‐Ray detectors by four times, and demonstrated a detectable X‐Ray dose rate as low as 0.5 μGy_air_ s^−1^. In addition to their previous work, the same research group has demonstrated the successful growth of high‐quality MAPbI_3_ single crystals with modulated growth rates using 3‐(Decyldimethylammonio)‐propane‐sulfonate inner salt (DPSI) as a regulator.^[14b]^ The Pb^2+^‐anchored DPSI ligands were found to be effective in reducing nucleation density and regulating ion movement to the crystal surface, resulting in improved crystal quality. These high‐quality crystals were employed as X‐ray detectors (Figure 10d). Notably, compared to the single crystals grown without DPSI modulation, the devices with DPSI exhibited stable and ultralow dark current densities (approximately 120 nA cm^−2^ @ 270 V cm^−1^), as demonstrated in Figure 10e. These devices demonstrated a high sensitivity of 2.9 × 10^6^ μC Gy_air_cm^−2^ and the lowest detectable dose rate of 5.7 nGy s^−1^ under 60 kVp irradiation (Figure 10f).

Additionally, perovskite bulk single crystals have been employed for the detection of γ‐rays, which possess higher energy and greater penetrating ability compared to X‐rays. Thanks to their strong stopping power and high linear attenuation coefficients toward γ‐rays, perovskites act as promising candidates for γ‐ray detection^[^ ^123^ ^]^ For instance, He et al. utilized the Bridgeman method to grow large‐scale CsPbBr_3_ single crystals with ultralow impurity. These crystals were then sliced into suitable slices for device fabrication, with the structure of Au/CsPbBr_3_/Ga (Figure 10g).^[50c]^ The asymmetrical electrodes of Au and Ga were deposited onto opposite surfaces of the crystal to efficiently collect charges and suppress device noise, resulting in high energy resolution of 3.9 and 3.8% in 122 keV ^57^Co (Figure 10h) and 662 keV ^137^Cs (Figure 10i) gamma‐rays, respectively.^[50c]^ Moreover, high‐quality 2D/3D heterojunctions^[^ ^124^ ^]^ and 2D bulk single crystals^[^ ^125^ ^]^ have also been extensively investigated for their high‐energy detection potentials. These structures exhibited high light yield and fast response, comparable to commercial CsI: Tl detectors, highlighting the potential of perovskite bulk crystals for high‐energy detection and imaging applications.

Beyond photodetectors, bulk crystals have been extensively investigated as promising materials for high‐performance solar cells with free grain boundaries and low defect levels. However, achieving high efficiency has proven to be challenging. Dong et al. reported a limited photovoltaic response in their Au/MAPbI_3_/Ga solar cell based on a 3 mm thick MAPbI_3_, where the thick active layer hindered the collection of photo‐induced charge carriers and resulted in high ohmic losses arising from the high intrinsic resistance.^[4a]^ In another study, they investigated the performance of a millimeter‐scale (thickness) lateral structure (Au/MAPbI_3_/Au), but only achieved a low efficiency of 1.88%.^[^ ^126^ ^]^ To address these issues, SCTFs with efficient carrier transport and extraction through hole and electron transport layers have been proposed. In the following section, we will summarize the progress and advancements in SCTF devices.

### 4.2 Single Crystal Thin Film Devices

The primary materials utilized to construct perovskite SCTF solar cells are MAPbBr_3_ and MAPbI_3_. Initially, Peng et al. developed a MAPbBr_3_ SCTF solar cell in a P–N junction configuration of FTO/TiO_2_/PVK/Au, achieving a PCE of 6.53%.^[^ ^127^ ^]^ However, there was a significant drop in the EQE. By adjusting the electron and hole transport layers to create a more suitable energy band structure, the PCE of the MAPbBr_3_ solar cell was improved to 7.11%, and it exhibited good stability compared to its polycrystalline counterparts.^[^ ^64^ ^]^ In comparison to MAPbBr_3_, which has a bandgap of around 2.3 eV, MAPbI_3_ exhibits a smaller bandgap of approximately 1.6 eV, making it a favorable candidate for high‐efficiency solar cells due to its more suitable absorption range. Zhao et al. reported a PCE of 8.87% for MAPbI_3_ solar cells based on the FTO/TiO_2_/MAPbI_3_/Spiro‐OMeTAD/Ag structure.^[^ ^128^ ^]^ However, the limited PCE was attributed to a low fill factor (FF) and small open‐circuit voltage (V_oc_), primarily caused by a thick absorption layer (≈15 μm) leading to strong interfacial carrier accumulation.^[^ ^129^ ^]^

**Figure 11:**
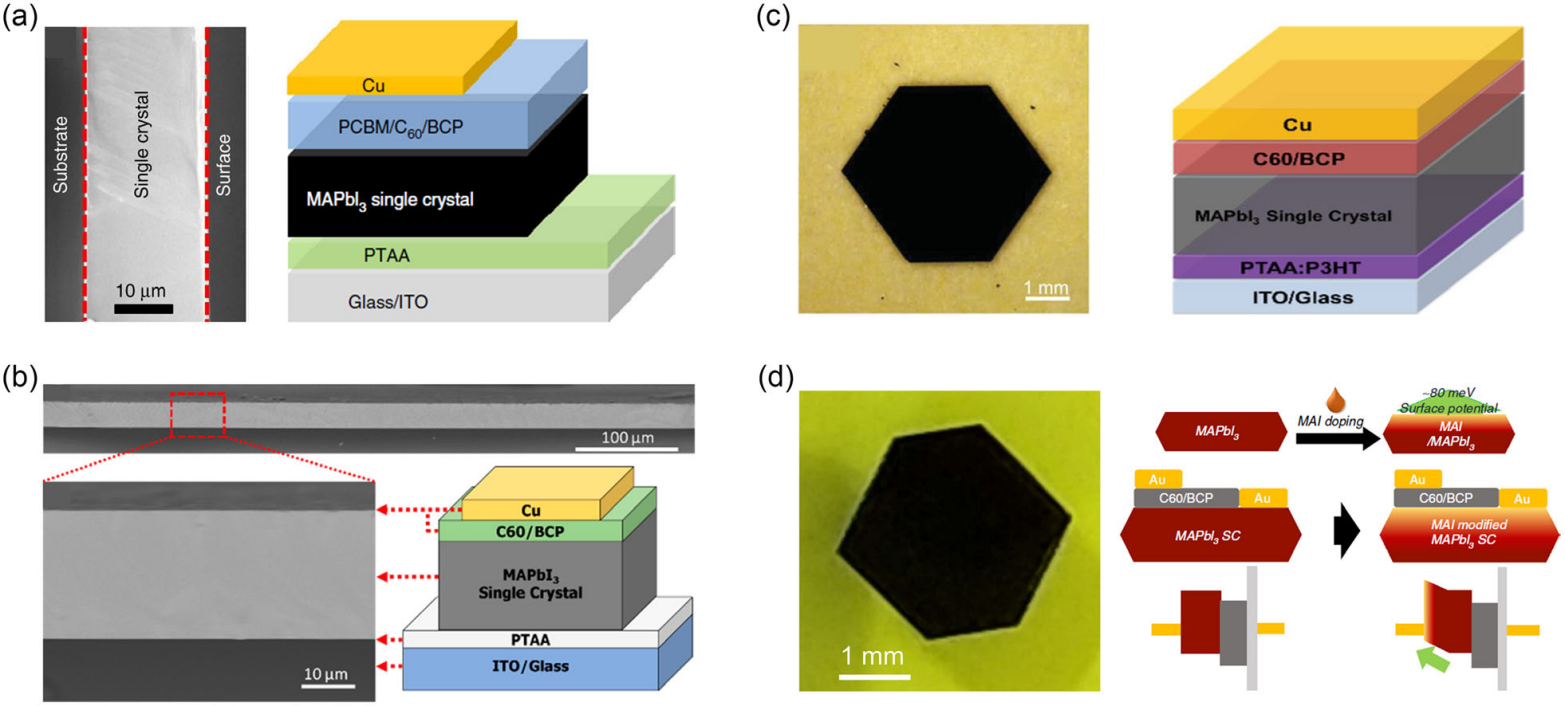
Solar cells based on perovskite SCTFs. a) Cross‐sectional SEM image of a MAPbI_3_ SCTF fabricated by using the hydrophobic interface confined lateral growth method and schematic of the device structure. Reproduced under the terms of the CC‐BY Creative Commons Attribution 4.0 International license (https://creativecommons.org/licenses/by/4.0).^[^ ^21^ ^]^ Copyright 2017, The Authors. Published by Springer Nature. b) Cross‐sectional SEM image of the FA_0.6_MA_0.4_PbI_3_ SCTF and the schematic of the device structure. Reproduced with permission.^[^ ^83^ ^]^ Copyright 2020, the American Chemical Society. c) Photograph of a MAPbI_3_ SCTF on PTAA: P3HT covered substrate and the configuration of the solar cell device. Reproduced with permission.^[^ ^130^ ^]^ Copyright 2021, John Wiley and Sons. d) Photograph of a MAPbI_3_ SCTF and the schematic of the lateral structure solar cell. Reproduced under the terms of the CC‐BY Creative Commons Attribution 4.0 International license (https://creativecommons.org/licenses/by/4.0).^[^ ^136^ ^]^ Copyright 2020, The Authors. Published by Springer Nature.

To overcome this issue, a thinner MAPbI_3_ SCTF (≈10 μm) was prepared using the space‐confined method with two ITO glass substrates covered with PTAA, as shown in **Figure** **11**a.^[^ ^21^ ^]^ By incorporating a solar cell structure of ITO/PTAA/MAPbI_3_/PCBM/C60/BCP/Cu, a PCE of 17.8% was achieved, representing a significant improvement over previous studies. The enhanced efficiency can be attributed to the thinner active layer of MAPbI_3_, which enables more efficient charge carriers collection and reduces recombination rates. Moreover, the use of lower temperatures (<90 °C) during the fabrication process restricts the loss of MAI and facilitates the formation of higher‐quality MAPbI_3_ thin films, contributing to the overall improved performance of the solar cells. As depicted in Figure 11b, a solar cell of a similar structure with a high‐quality MAPbI_3_ active layer demonstrated a PCE of 21.9%, and a high FF of 83.5% indicating efficient carrier transport dynamics and strong build‐in fields.^[^ ^83^ ^]^ Furthermore, interface engineering has been applied to reduce trap densities and nonradiative recombination. In a study by Li et al., poly(3‐hexylthiophene) (P3HT) was incorporated into PTAA to enhance the hydrophobicity of the substrate, suppress undercoordinated Pb^2+^ species, and improve ion diffusion rates in the solution.^[^ ^130^ ^]^ This approach successfully reduced both interface and bulk defect densities in the MAPbI_3_ SCTF, resulting in a PCE of 22.1% and a *V*_oc_ of 1.13 V (Figure 11c). These results represent the highest values reported to date for MAPbI_3_ single‐crystal solar cells.

In addition to the strategies mentioned above, bandgap engineering techniques have shown promise for improving solar cell efficiency by expanding the absorption range to match the ideal bandgap of approximately 1.4 eV, as dictated by the Shockley–Queisser limit.^[^ ^131^ ^]^ While the α‐FAPbI_3_ possesses an ideal bandgap for high‐efficiency solar cells, its stability at room temperature is limited, and it tends to transform into a nonperovskite β‐phase. To address this issue, a suitable approach is to mix the FA cation with the MA cation, which effectively reduces the bandgap of MA‐based perovskite. For instance, a solar cell with the structure of ITO/NiO_*x*_/(FAPbI_3_)_0.85_(MAPbBr_3_)_0.15_/TiO_2_/Ag was reported to achieve a PCE of 12.18% with an active layer thickness of 24.5 μm.^[^ ^132^ ^]^ To further reduce the bandgap, FA_0.6_MA_0.4_PbI_3_ single crystals were prepared by mixing FAPbI_3_ with MAPbI_3_, resulting in a reduced bandgap of 1.48 eV and a remarkable PCE of 22.9%.^[^ ^133^ ^]^ Recently, Almasabi et al. utilized the same active layer (FA_0.6_MA_0.4_PbI_3_) and introduced a novel HTL of PTAA with {SAM, [2‐(3,6‐dimethoxy‐9H‐carbazol‐9‐yl)ethyl]phosphonic acid), (MeO‐2PACz)}. The MeO‐2PACz SAM improved the mechanical adhesion between the perovskite and the substrate, resulting in the creation of inverted solar cells that exhibited significantly improved operational stability.^[^ ^134^ ^]^ Remarkably, this device achieves the highest reported PCE of 23.1% among all single‐crystal perovskite solar cells.

Compared to vertically structured solar cells, lateral structures exhibit reduced thickness‐induced intrinsic resistance. Lee et al. employed a space‐confined method to prepare wafer‐scale MAPbI_3_ SCTFs, which were then fabricated into lateral solar cells with PCBM/Ag and Au electrodes on the same surface.^[^ ^135^ ^]^ However, due to gaps between the single crystalline stripes, the achieved PCE was limited to 4.83%. Subsequently, Hung's group improved upon this by preparing large‐scale free‐standing MAPbI_3_ SCTF using the surface tension assistant method and designing solar cells with Au/BCP/C_60_ and Au electrodes on the MAPbI_3_ surface, resulting in a PCE of 5.9% under 0.25 sun illumination.^[^ ^71^ ^]^ To further enhance the efficiency of lateral structural solar cells, the same group prepared MAPbI_3_ SCTF and passivated surface defects with MAI to address the tendency of MAI escaping during the high‐temperature growth process, as illustrated in Figure 11d.^[^ ^136^ ^]^ The resulting solar cell, utilizing the same electrodes as the previous work, achieved a PCE of 11.2%, surpassing all other reported perovskite single‐crystal lateral solar cells. Despite this achievement, the development of lateral structural solar cells is still limited by challenges related to insufficient charge extraction and a limited active range.

In addition to their application in solar cells, SCTFs have also demonstrated remarkable performance in field‐effect transistors (FETs) and photocatalysts. FETs, with their requirements for lateral and interfacial transport, are particularly susceptible to surface contamination and defects commonly found in polycrystalline films and bulk single crystals. Yu et al. fabricated MAPbX_3_ SCTFs with a thickness of 2.5 μm and ultrasmooth surface (sub‐nanometer roughness) using the space‐confined method.^[^ ^137^ ^]^ The FETs based on these films exhibited high field‐effect mobility of up to 4.7 cm^2^ V^−1^ s^−1^ in p‐channel devices and an on‐off ratio of up to 10^5^.^[^ ^137^ ^]^ Liu et al. conducted a study on (BA)_2_(MA)_*n*‐1_Pb_n_I_3*n*+1_ single crystal FETs and observed n‐type transistor behavior as well as a significant on‐off ratio when ion migration was suppressed at lower temperatures.^[^ ^138^ ^]^ Besides, SCTFs have also shown impressive performance in photocatalysts. Wang et al. achieved significant improvements in the photoelectrochemical performance of MAPbBr_3_ SCTFs by surface passivation using a thin layer of Al_2_O_3_, resulting in a nearly fivefold enhancement.^[^ ^139^ ^]^ This surface passivation effectively suppressed trap‐assisted recombination, leading to improved photocatalytic performance.

Perovskites are also good candidates for light emitting applications, particularly as laser gain media, due to their advantageous properties such as high absorption coefficients, high PL quantum yield, and weak Auger recombination. Zhang's group has successfully demonstrated several examples of lasing, including CsPbBr_3_ microplate/nanowire lasing[^10^, ^75^] and 2D perovskite thin film lasing.^[^ ^57^ ^]^ Nguyen et al. reported lasing in a MAPbBr_3_ SCTF (≈3.6 μm in thickness) sandwiched between two DBR substrates, achieving a low threshold of 26 μJ cm^−2^.^[^ ^140^ ^]^ Similarly, other works of MAPbBr_3_ SCTFs sandwiched into DBR substrates have been reported, exhibiting low thresholds (≈4 μJ cm^−2^)^[^ ^22^ ^]^ and single mode^[^ ^141^ ^]^ lasing with quality factor (*Q* factor) of ≈1,286 after careful optimization of growth and measured conditions. These results highlight the potential of SCTFs as promising gain media for lasers. Besides, surface patterning of single crystal surfaces can be employed to tune lasing behaviors, resulting in interesting lasing phenomena, which will be further discussed in the next section.

### 4.3 Devices Based on Surface‐Patterned Single Crystals

**Figure 12:**
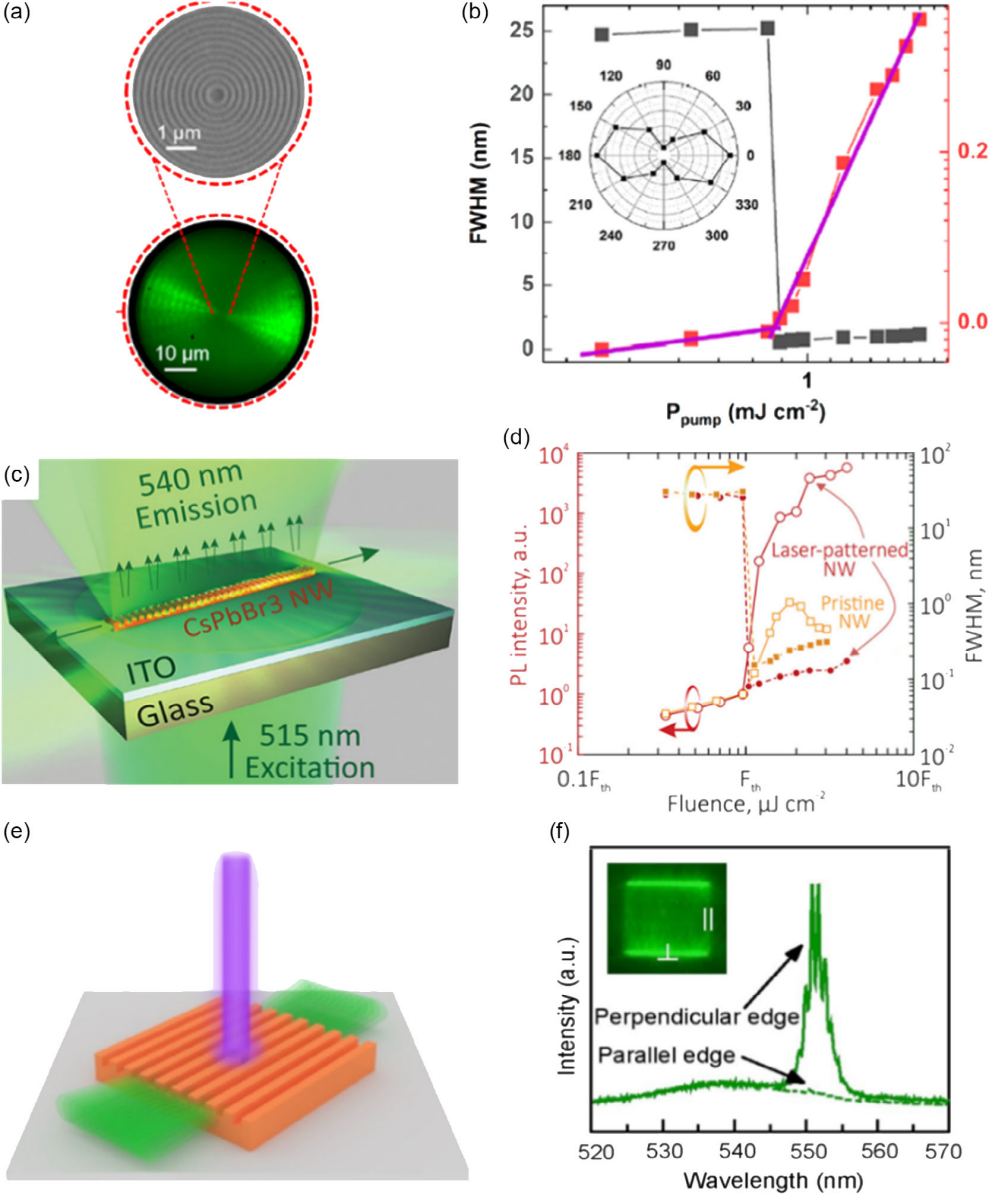
Lasers based on surface‐patterned perovskite single crystals a) SEM and micrograph of MAPbBr_3_ microplate with surface circle grating fabricated by FIB. b) FWHM and output intensity change as a function of excitation intensity, with the output polarization given in the inset. a,b) Reproduced with permission.^[^ ^142^ ^]^ Copyright 2021, the American Chemical Society. c) Schematic showing the laser‐patterned (period = 260 nm) CsPbBr_3_ nanowire emitting from its surface upon laser pumping. d) Power‐dependent PL intensity and FWHM of the pristine nanowire and the laser patterned nanowire. c,d) Reproduced with permission.^[^ ^94^ ^]^ Copyright 2020, the American Chemical Society. e) Schematic of a pump laser and the lasing emission from the surface patterned MAPbBr_3_ microplate. f) Micrograph of lasing emission from a surface patterned MAPbBr_3_ with 90° gratings. e,f) Reproduced with permission.^[^ ^111^ ^]^ Copyright 2022, John Wiley and Sons.

Surface‐patterned perovskite single crystals provide a means to control light−matter interactions through predesigned optical structures such as gratings (**Figure** **12**a).^[^ ^142^ ^]^ Liu et al. successfully designed highly ordered circular grating arrays on the surface of MAPbBr_3_ microplates using FIB, enabling more homogeneous light−matter interactions.^[^ ^142^ ^]^ Consequently, single‐mode lasers with highly coherent beams were obtained, demonstrating a 4.5 times higher *Q* factor (≈1,250) and eight times higher PL intensity compared to their polycrystalline counterparts. Figure 12b illustrates the logarithmic S‐shaped profile curve of a line grating laser (with a period of 270 nm) as the irradiation power changes, indicating a threshold of approximately 0.9 mJ cm^−2^ and optical polarization perpendicular to the periodic direction. Wang et al. employed the same method to pattern the surface of CsPbBr_3_ microplates, achieving colorful patterns and the ability to tailor the lasing behavior in situ, showcasing the promise for the controlled design of light sources and the on‐chip integrated devices.^[^ ^93^ ^]^ Laser ablation was adopted to create gratings on the surface of the CsPbBr_3_ nanowire, as depicted in Figure 12c, enabling stimulated emission in the vertical direction of the nanowire.^[^ ^94^ ^]^ As illustrated in Figure 12d, compared to pristine nanowires, the surface patterned CsPbBr_3_ nanowire exhibited a twofold increase in PL intensity under the same excitation conditions, with a significantly reduced full‐width at half maximum (FWHM) and a high *Q* factor of up to 6,000, indicating that the surface patterning process does not degrade much the quality of the nanowire but effectively tunes the output direction of the lasers.^[^ ^94^ ^]^

Additionally, the solution imprinting method was also used to create patterns on the surface of perovskite single crystals, as schematically illustrated in Figure 12e. This method allowed for the lasing beam emission from specific edges of a surface patterned MAPbBr_3_ microplate with line gratings. As shown in Figure 12f, the emission intensity from the perpendicular edge of the MAPbBr_3_ microplate with 90° gratings was significantly higher than that from the parallel edge. This observation suggests that the preferential emission direction can be tuned by adjusting the angle between grating lines and one edge of the microplate.^[^ ^111^ ^]^ Based on that, a directional single‐mode laser with a threshold of 15.1 μJ cm^−2^ and a *Q* factor exceeding 1,000 was achieved.

Last but not least, numerous passivation approaches have been employed to enhance perovskite material stability and device lifetimes by reducing defect density, enhancing photoelectric properties, and optimizing carrier dynamics, photocurrent, and band gaps. Since PMMA, 2D perovskite, trioctylphosphine oxide (TOPO), and fullerene were utilized for passivation on polycrystalline thin films,^[^ ^143^ ^]^ several effective passivation techniques have been successfully applied to single crystal perovskites. At elevated temperatures, there is a risk of MAI escaping from the surface of MAPbI_3_ single crystals, resulting in increased surface carrier recombination and the formation of defects, which adversely affect the performance of solar cells. To address this issue, Chen et al. employed MAI passivation on MAPbI_3_ SCTFs and significantly improved the PCE of MAPbI_3_ SCTF solar cells to 17.8%.^[^ ^21^ ^]^ Song et al. introduced a thin layer of MAI on the lateral solar cells of MAPbI_3_ SCTF to optimize the anode contact, where the passivation with MAI led to a notable shift in surface potential toward the valence band, enhancing the V_OC_ and FF of the solar cells.^[^ ^136^ ^]^ Moreover, Chen et al. introduced a surface passivation technique for addressing vacancy defects in MAPbBr_3_ single crystals through immersing in MABr and PEABr solutions.^[^ ^144^ ^]^ This passivation process, coupled with limited ion migration, resulted in improved PL lifetime and enhanced X‐ray device performance. Beyond precursor treatments, alternative molecular passivation methods have also been explored. Guo et al. utilized 3‐mercaptopropyl (dimethoxy) methylsilane (MDMS) to coordinate with Pb^2+^ and mitigate surface defects, resulting in a remarkable enhancement of the PCE of MAPbI_3_ solar cells to 22.2%, the highest value for MAPbI_3_ based single‐crystal solar cells.^[^ ^145^ ^]^ Notably, the incorporation of MDMS effectively impeded surface ion migration, leading to enhanced reverse‐bias stability.

## 5 Summary and Outlook

In conclusion, we have provided an overview of recent advancements in the preparation of large‐scale perovskite single crystals and surface patterning technologies. We have also discussed various efficient optoelectronic applications based on these single crystals with/without surface patterns. The advantages of perovskite single crystals can be summarized into three aspects. First, bulk single crystals serve as ideal platforms for intrinsic and fundamental studies of perovskite materials, as well as for high‐performance photodetector arrays including low‐energy and high‐energy photodetection and imaging. Second, the SCTFs have demonstrated more impressive performance compared to bulk single crystals in low‐energy photodetection with high responsivity and solar cells with high PCE of greater than 23%. Moreover, the surface patterns can modulate and enhance the light‐matter interactions, providing promising structures for optoelectronic and/or optical devices. These achievements in material preparations and device performance meet the basic requirements for future commercialization, facilitating the development of perovskite in photovoltaic, optoelectronic, and on‐chip optical/electronic technologies.

Although tremendous achievements and encouraging progress have been made by the single crystal growth and surface patterning technologies, there are still some challenges:

The quality of perovskite single crystal quality still requires improvement. While perovskite single crystals exhibit low trap densities, there are challenges associated with organic–inorganic hybrid perovskite crystals grown from solutions. These crystals tend to possess high surface trap densities, which can be attributed to the loss of organic ions during growth because of the different solubilities of organic and inorganic ions. To address this issue, it is recommended to explore the use of mixed solvents or new solvents that can decrease the crystallization temperature. Lowering the temperature during crystallization has been proven to limit the escaping of organic ions and reduce the surface defect density. Furthermore, the presence of residual solvent molecules inside the crystals can lead to the formation of new trap sites, thereby increasing trap densities and accelerating aging. It is therefore important to optimize growth conditions and explore various passivation techniques to improve single‐crystal quality and reduce surface traps.
While the majority of research in the field of perovskite single crystals has focused on organic–inorganic Pb‐based materials, including MAPbI_3_, MAPbBr_3_, MAPbCl_3_, etc., it is important to address the potential challenges associated with the unstable organic components and the toxic nature of the lead element. Consequently, there is a growing need to explore the development of all‐inorganic and Pb‐free perovskite single crystals.
The current thickness of SCTFs, approximately 10 μm, remains too large for vertical structure devices. Although single‐crystal solar cells have achieved a PCE exceeding 23%, there is still a notable disparity between their performance and the theoretically predicted efficiency of over 30%. To enhance PCE, it is essential to reduce the thickness of SCTFs to alleviate intrinsic resistance and bolster charge carrier extraction. Addressing the challenges related to the thickness and quality of single crystals requires a simultaneous approach, thus the development of novel additive materials becomes crucial. Moreover, utilizing thin films with mixed A‐site cations, such as FA and MA, can effectively narrow the bandgap, extend the absorption range, and ultimately improve solar cell performance. Additionally, there is room for optimization in the device structures of both vertical and lateral single‐crystal solar cells. It has been demonstrated that the design of lateral structured devices extends beyond the simple deposition of two symmetrical electrodes directly onto single crystals. By introducing different HTLs and ETLs beneath the electrodes, the charge transport properties can be effectively adjusted.
During the preparation of integrated perovskite with substrates, interfacial engineering can effectively mitigate the lattice mismatch between the perovskite and the substrate. The residual strain resulting from the mismatch can lead to poor contact with the electrodes and promote recombination at the interface. Therefore, detailed investigations at an atomic level and the utilization of methods to relieve the lattice strain are crucial, such as designing a buffer layer between the perovskite and substrate, as well as modifying the substrate surface using molecules.
The utilization of top‐down technologies in perovskite surface patterning, such as FIB, EBL, and laser ablation, can often result in inevitable damage and high costs. Therefore, it is necessary to explore methods that can effectively passivate and/or heal damaged regions. Moreover, the development of rapid fabrication techniques that can reduce costs is of great value and should be further investigated.
Achieving controllable and precise patterning using the bottom‐up method remains a significant challenge, particularly with the one‐step nanoimprinting method. To overcome this issue, the utilization of multiprocesses, such as employing prepatterned substrates as templates, can help localize the precursor and nucleation positions.

## Conflict of Interest

The authors declare no conflict of interest.
